# Adaptation and resilience of commercial fishers in the Northeast United States during the early stages of the COVID-19 pandemic

**DOI:** 10.1371/journal.pone.0243886

**Published:** 2020-12-17

**Authors:** Sarah Lindley Smith, Abigail S. Golden, Victoria Ramenzoni, Douglas R. Zemeckis, Olaf P. Jensen

**Affiliations:** 1 Department of Human Ecology, Rutgers University, New Brunswick, New Jersey, United States of America; 2 Department of Marine and Coastal Sciences, Rutgers University, New Brunswick, New Jersey, United States of America; 3 Graduate Program in Ecology and Evolution, Rutgers University, New Brunswick, New Jersey, United States of America; 4 Department of Agriculture and Natural Resources, Rutgers University, Toms River, New Jersey, United States of America; 5 Center for Limnology, University of Wisconsin-Madison, Madison, Wisconsin, United States of America; University of New Haven, UNITED STATES

## Abstract

Commercial fisheries globally experienced numerous and significant perturbations during the early months of the COVID-19 pandemic, affecting the livelihoods of millions of fishers worldwide. In the Northeast United States, fishers grappled with low prices and disruptions to export and domestic markets, leaving many tied to the dock, while others found ways to adapt to the changing circumstances brought about by the pandemic. This paper investigates the short-term impacts of the early months of the COVID-19 pandemic (March-June 2020) on commercial fishers in the Northeast U.S. to understand the effects of the pandemic on participation in the fishery and fishers’ economic outcomes, using data collected from an online survey of 258 Northeast U.S. commercial fishers. This research also assesses characteristics of those fishers who continued fishing and their adaptive strategies to the changing circumstances. Analysis of survey responses found the majority of fishers continued fishing during the early months of the pandemic, while a significant number had stopped fishing. Nearly all reported a loss of income, largely driven by disruptions of export markets, the loss of restaurant sales, and a resulting decline in seafood prices. Landings data demonstrate that while fishing pressure in 2020 was reduced for some species, it remained on track with previous years for others. Fishers reported engaging in a number of adaptation strategies, including direct sales of seafood, switching species, and supplementing their income with government payments or other sources of income. Many fishers who had stopped fishing indicated plans to return, suggesting refraining from fishing as a short-term adaptation strategy, rather than a plan to permanently stop fishing. Despite economic losses, fishers in the Northeast U.S. demonstrated resilience in the face of the pandemic by continuing to fish and implementing other adaptation strategies rather than switching to other livelihoods.

## 1. Background

### 1.1 Introduction

Commercial fishers and the commercial fishing industry were forced to confront myriad challenges brought about by the COVID-19 pandemic, including the resulting social distancing requirements and economic crisis [1]. These factors, individually and cumulatively, tested the resilience of fisheries systems. For many of the estimated 260 million people worldwide who make a living from global marine fisheries, including as harvesters and within the seafood sector [2], the pandemic endangered their livelihoods and highlights their vulnerability to the social and economic effects of COVID-19 [3]. As social-ecological systems, fisheries often demonstrate both adaptation to changing ecological or socio-economic drivers, and resilience, or the ability of the system to absorb and adapt to disturbances while maintaining the same structure [4, 5]. In times of change and uncertainty, resilient systems are those that will be able to weather an adverse event and persist, including through adaptation mechanisms, while non-resilient systems may experience catastrophic change or an irreversible shift to another state [4, 6]. Understanding fishers’ ability to adapt to change (i.e., adaptive capacity), including disruptions caused by the COVID-19 pandemic, can help to guide recovery efforts for fisheries in times of crisis or rapidly changing circumstances [7], and promote resilience for fisheries, enabling them to endure this and future crises. To that end, this article explores the immediate socioeconomic impacts of the COVID-19 pandemic on commercial fishers in the Northeast U.S. (from Maine through North Carolina) along with adaptation strategies and responses to this unprecedented situation.

Throughout the early months of the COVID-19 crisis, fisheries globally experienced severe impacts from the pandemic and its associated restrictions, with many fishing fleets experiencing a substantial decline in activity for the early part of 2020 [1, 3, 8]. The pandemic caused significant disruptions to food supply chains around the world [9], and seafood supply chains were no exception [1]. Industrial fleets in China, France, Spain, and Italy, for example, saw a 40–50% decrease in fishing effort in the first quarter of 2020 compared with the previous year [1].

In the United States, as in much of the rest of the world, fishing is considered an essential business, and was allowed to continue through the early months of the pandemic while stay-at-home orders were in place in many states. However, social distancing measures and the resulting economic recession greatly shifted demand for seafood. In the spring of 2020, the pandemic had a significant impact on consumer demand for American seafood products, and subsequently the price of many seafood products dropped precipitously, either because they relied on export markets which were severely disrupted, or because they relied to a large extent on restaurant sales [1, 3]. As early as January 2020, there were reports that the COVID-19 pandemic and its repercussions in China were impacting demand for U.S. lobster exports [1], the demand for which has already been in decline due to tariffs imposed by China in 2018 [10], and impacts on the demand for many other seafood products soon followed.

In studying trends in import and export data, White et al. [8] found a significant decline for the U.S. in both imports and exports as of March 2020, and observed that exports of live, fresh, or chilled seafood had declined much more sharply than for frozen seafood products. Conversely, in winter and early spring of 2020, both foreign and domestic demand for frozen seafood remained high [8], and the FAO reported that effects on retail sales were mixed, as households looking to stock up on non-perishable goods increased their purchases of canned and frozen seafood products [11]. Along with a declining export market, seafood dealers also faced challenges with freight costs and flight availability to transport seafood overseas [1, 12].

Prior to the outbreak of the pandemic, 70% of seafood spending in the United States occurred in restaurants [13]. These restaurant sales were severely reduced or in some cases lost altogether for the duration of stay-at-home orders when restaurants were closed or limited to take-out [9], and were slow to return as stay-at-home orders were lifted but restaurants remained closed or were limited to outdoor dining or serving at reduced capacity. In addition, there were significant disruptions to the supply chain from challenges related to social distancing requirements in processing facilities, at seafood dealers, at retailers, and with seafood transportation, as there have been in supply chains throughout the world. Because of the nature of seafood, which spoils quickly and requires freshness to meet market demand, supply chain disruptions can result in seafood being discarded. Frequently, fishers and processors were freezing and storing catch, or selling more frozen seafood than pre-pandemic, due in part to increasing demand for frozen seafood at retail outlets [14]. However, there were concerns about whether there would be a sufficient market for these frozen products once restrictions eased [15], and some processors were running out of freezer space to store frozen seafood products.

Along with challenges related to the marketing and sales of seafood were challenges related to fishing itself. Many fisheries require fishers to go offshore on multi-day trips with multiple crew members, presenting challenges when social distancing is necessary, and potentially providing opportunities for outbreaks of COVID-19 to occur. Some fishers were reducing the number of crew they are taking out to sea to facilitate social distancing or to reduce the number of people among whom they needed to split revenue, which may have required them to fish closer to shore or on shorter trips, or resulted in safety concerns if they were trying to do the same work with a smaller crew. Shoreside support services, such as fuel, ice, and repairs, were also sometimes affected by social distancing requirements, and in some cases may not have been open in the early months of the pandemic. Fishing communities, particularly in other parts of the United States and the world where fishers migrate to a fishing community for a particular season, posed the possibility of becoming hotspots for COVID-19 [3]. In the United States there were instances of local contagion on factory trawlers or in fish processing plants [16, 17]. In summary, the early months of the pandemic posed a suite of challenges for fishers that threatened their businesses, giving them the option to either continue fishing while suffering significant economic losses, or to tie up their boats and leave fishing for the time-being or altogether.

Despite these challenges, there were also numerous examples of fishers and fishing communities working to adapt to the COVID-19 pandemic. Local food networks, state- and locally-led marketing initiatives, and community-supported fisheries provided new or increasing opportunities for fishers to sell their catch, helping fishing businesses to stay afloat, and filling some of the gaps left by market disruptions through allowing consumers to access local seafood [3]. The state of Rhode Island created a new permit to allow for fishermen to sell finfish directly to consumers along with lobsters and crabs, whereas previously finfish were required to be sold by a dealer to meet food safety requirements [18]. Other states and communities also engaged in efforts to market seafood to local customers or to connect them to fishers conducting direct sales to make up for some of the shortfall in revenues [18–20]. Media outlets reported an increased interest in cooking seafood at home [14], and those who operate community-supported fisheries and other ventures bringing local seafood direct to consumers found an increased interest in supporting local fishers and buying locally-caught seafood, including significant growth in participation in seafood subscription services [1, 21]. These findings mirror a trend seen across food systems of increasing interest in locally-produced food items in the early days of the pandemic [22]. However, these adaptations are not likely to make up for the significant loss of domestic and export markets, particularly for high-value, high-volume species like the American lobster (*Homarus americanus*) or Atlantic sea scallop (*Placopecten magellanicus*) in the Northeast U.S.

### 1.2 Resilience of fisheries systems

Promoting resilience of fisheries as social-ecological systems means retaining the essential functions of the system while responding to shocks and disturbances. For commercial fisheries in the Northeast U.S., these essential functions include sustaining food production, fishing jobs, identities, and cultures, as well as promoting resilient and healthy marine resources and ecosystems [23]. Fishers and fishing communities are no strangers to challenges and disaster, having been affected by natural and man-made disasters including oil spills, hurricanes, floods, and climate change. Each of these can result in impacts to fish stocks, infrastructure, fishing communities, and markets, and all are further exacerbated by climate change [24].

The Northeast U.S. region contains some of the most valuable fisheries in the United States, including Atlantic sea scallop and American lobster, along with some of the nation’s oldest commercial fisheries [25]. Many coastal communities in the region have strong economic and cultural ties to fishing [26, 27]. However, many fisheries and fishing communities in the Northeast have suffered recent shocks related to trade policy and other economic factors, overfishing, natural disasters, climate change, and other drivers [28–30]. For example, commercial fisheries in New York and New Jersey were hard hit by Hurricane Sandy and its aftermath in 2012 and were subsequently declared federal fishery disasters later that year [31, 32]. The Northeast multispecies (groundfish) fishery was declared a federal disaster multiple times in the 1990s by the National Oceanic and Atmospheric Administration (NOAA), and more recently received a federal disaster declaration for the years 2011–2013 after significant cuts were made to the catch limits of many species following a failure to rebuild several stocks, including Atlantic cod (*Gadus morhua*), dramatically affecting participating fishers [31]. This fishery has a long history of overexploitation dating back several decades [33]. More recently, despite being a well-managed fishery with record high landings over the last decade [33], the New England lobster fishery has suffered from low prices and restricted markets. These have resulted from seafood tariffs from China of up to 40%, leading to a 65% decrease in lobster exports to China in 2019 [8]. Additionally, most fishers have experienced changes to their livelihood from the creation and continued revision of fisheries regulations and management regimes, some of which result in dramatic impacts to their ability to pursue their livelihoods [34, 35]. Each of these experiences provides context for understanding the impacts of the COVID-19 pandemic on commercial fishers, and how they may respond to the new pandemic-driven challenges they face.

Evidence regarding the effect of previous disturbance on the ability of social-ecological systems to withstand future shocks is mixed. Some studies suggest that experience with past disasters and other social and ecological threats to local livelihoods may increase a community’s resilience to deal with future disasters [36, 37]. As fishers weather the challenges of previous events, many may be able to emerge with their livelihoods intact and a higher confidence in their ability to remain fishing [7]. On the other hand, some fishers may exhibit increased vulnerability to such an event, experiencing the COVID-19 pandemic as an additional disaster, compounding on existing trauma resulting from extreme weather, policy changes, and economic downturns [38]. As fishers and fishery systems experience and adapt to these events, they may become increasingly vulnerable to other types of disturbances [39]. For example, it has been reported that many fishers in the New England multispecies (groundfish) fishery have experienced moderate to severe psychological distress and social disruption as a result of fishery management actions and the associated economic impacts of persistent difficulties in rebuilding some depleted stocks [40]. The COVID-19 pandemic may exacerbate these existing challenges.

In a recent editorial, several authors argued for the need for researchers to investigate the social impacts of COVID-19 on fisheries [3]. While in the Northeast U.S., the effects of the pandemic may be cumulative, progressive, and linger over several years, it is imperative to gain a better understanding of how fishers have been affected by the pandemic in the short-term to inform ongoing management and mitigation policies. Such an understanding must consider the immediate economic effects of epidemiological measures on fishing activities in the Northeast U.S., how fishermen have responded to direct and indirect effects of social distancing, whether fishers decided to continue fishing during the pandemic or to exit the fishery altogether, and how and in what ways fishers are able to adapt to the rapidly changing circumstances. Most significantly, it is essential to identify how responses, coping mechanisms, and strategies can reshape and, in turn, bolster or constrain the adaptive capacity, and in turn the resilience, of fisheries and fishing communities in the Northeast U.S. to address future challenges.

To that end, this study seeks to answer the following questions:

- What were the immediate socioeconomic impacts of the COVID-19 pandemic on commercial fishers in the Northeast United States? Were these impacts experienced universally, or did they differ based on factors such as participation in particular fisheries or location?
- Which commercial fishers in the Northeast U.S. continued fishing during the pandemic, and what are the characteristics of those who remained fishing compared with those who stopped fishing?
- What were the most significant drivers of the pandemic’s impact on fishers?
- How did fishers adapt to the changing circumstances dictated by the COVID-19 pandemic?

## 2. Methods

### 2.1 Survey instrument and data collection

To understand the early impacts of the COVID-19 pandemic on the commercial fishing industry, we designed an online survey (S1 File) using the Qualtrics research software platform. The survey was disseminated to commercial fishers in the Northeast U.S. (Maine through North Carolina) by email via industry associations, state and federal agencies, two regional fishery management councils, and Extension agents (including Sea Grant and Cooperative Extension agents at state universities) to recruit participants. The survey was sent to all the fishery associations for which we could obtain contact information for to maximize diversity across fisheries and states. We also distributed this survey among state commercial fishing license holders in Maine through email. While this approach resulted in 49% of all survey respondents coming from Maine, it should be noted that Maine also has the largest proportion of commercial fishers in the Northeast U.S. (roughly 38% of the region’s commercial fishers [41]). In addition, this industry has been impacted considerably by the pandemic. We sent reminders to fishing associations, state and federal agencies, fishery management councils, and Extension agents about distributing the survey, but did not send reminder emails to individual fishers.

Survey responses were collected between May 14 and June 14, 2020. This period corresponded with the end of stay-at-home orders for most states included in the survey, as most of the stay-at-home orders began to be lifted after the survey had been distributed. (A list of the dates for which stay-at-home orders or advisories were in place for each state is in S1 Table). Thus survey responses reflect conditions in the early months of the pandemic, starting in mid-March of 2020 when the World Health Organization (WHO) first declared COVID-19 a pandemic and Northeast U.S. states were asking residents to stay at home while closing businesses and restaurants, as well as the period just following each state’s initial ‘re-opening’ when businesses such as restaurants were allowed to resume with restrictions. The survey was approved by Rutgers University’s Institutional Review Board (#Pro2019002753), and informed consent was obtained digitally from participants.

To assess socioeconomic impacts on commercial fishers, participants were asked a series of questions (S1 File) about changes to their fishing practices since the start of the COVID-19 pandemic. Survey respondents were asked to report on their fishing activity and impacts to their livelihood since the start of stay-at-home orders in their respective states. Questions included whether participants were still fishing; the fisheries they ordinarily participate in and what fisheries, if any, they were participating in during the early stages of the pandemic; impacts to their fishing income, landings, effort, and costs; the effect of certain pandemic-related impacts on their ability to go fishing; adaptations they have made to the circumstances created by the pandemic; and the ways in which the pandemic has affected their well-being and that of their family members. The survey included 51 questions, including several different modules containing both Likert-scale and open-ended questions, and took participants about 20–25 minutes to complete. This paper focuses on analysis of responses to a subset of the survey questions.

There are limitations inherent in using an online survey tool with fishers. Disseminating the survey through fishing organizations means the survey respondents may be skewed toward those fishers who are actively engaged in one or more industry organizations, or who follow correspondence from the fishery management councils or their local Extension agents. While the survey was targeted at owners, captains, and crew, survey respondents were expectedly made up largely of owners (n = 152) and captains (n = 81), with a smaller number of crew (n = 21), who are often underrepresented in surveys because they can be harder to reach [42]. As respondents were allowed to self-identify their role, and were not provided with specific definitions, there is likely to be some overlap between owners and captains, as many survey respondents, particularly those fishing on smaller vessels, are likely be owner/operators.

Survey responses were analyzed and visualized using the statistical programming language R version 3.6.0 [43]. Summary statistics were derived for demographic information. Pearson’s chi-squared tests and t-tests were used to evaluate the relationship between responses to key survey questions (e.g., whether respondents had continued fishing) and respondent attributes. Responses to Likert-scale survey questions were visualized using the ‘sjPlot’ package (version 2.8.3) in R [44]. Respondents’ fishery participation was visualized using the ‘sankeyNetwork’ function in the ‘networkD3’ package in R (version 0.4) [45]. Informal conversations with key stakeholders including fishery managers, scientists, and members of the fishing industry were also conducted to clarify and help interpret some of our survey findings and conclusions, including with members and staff of the New England Fishery Management Council, the Mid-Atlantic Fishery Management Council, NOAA Fisheries Northeast Fisheries Science Center, the Science Center for Marine Fisheries (SCEMFIS), and the Maine Department of Marine Resources (to learn more about impact to the lobster fishery specifically).

### 2.2 Fisheries data

In order to understand broader region-wide trends in fisheries landings and price, we received access to data on individual dealer-reported landings and price for seven federally-managed commercial fish species from NOAA Fisheries through July of 2020 and for recent pre-pandemic years (2015–2019) [46]. The data were subsequently aggregated and analyzed to compare recent landings and price trends for 2020 with previous years. Some of the fishermen surveyed would also have participated in state-managed fisheries. However, due to the variability of state license systems and the fact that fishers can possess licenses from more than one state, to avoid potential duplications we chose to analyze only federal data. The federal fisheries data we include provide some insight into landings and price trends in the region, but are not comprehensive of all of the region’s fisheries.

## 3. Results

### 3.1 Survey participation

**Fig 1:**
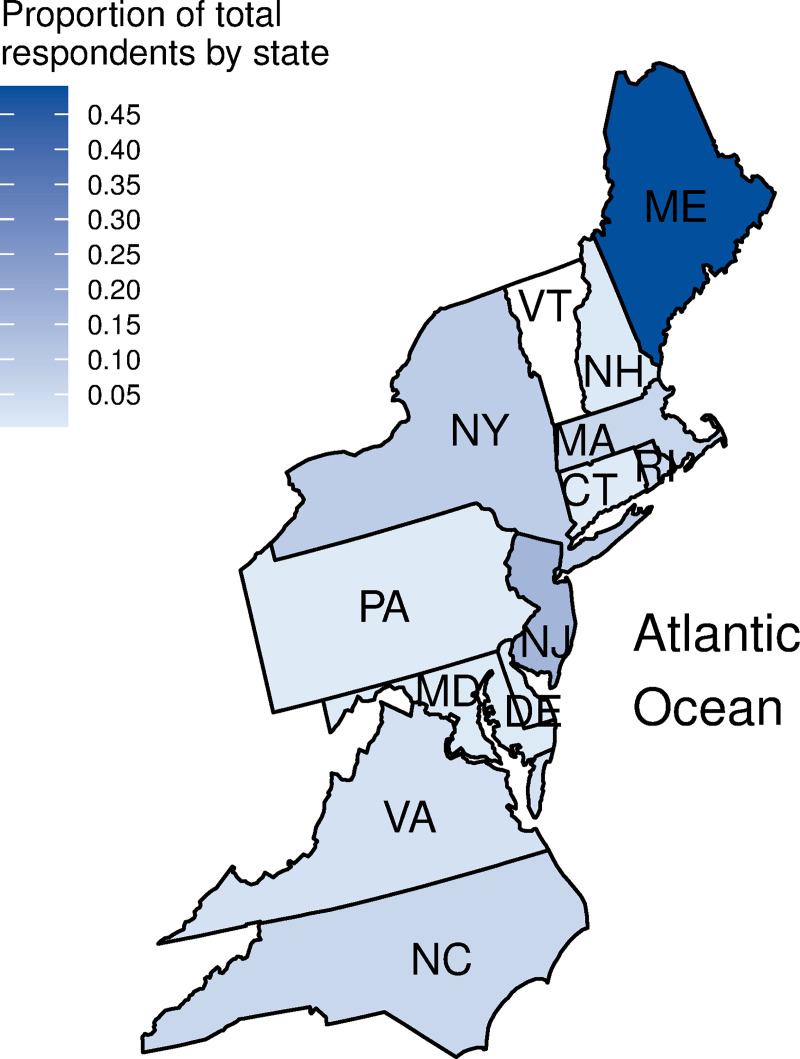
Proportion of total respondents by state. The map shows the Northeast U.S. states (Maine through North Carolina) with percentage of survey responses represented by the blue shading. Responses also included one vessel owner who lived in Florida but owned a boat fishing in the Northeast (not included in the figure).

Of 330 initial survey responses received, we received 258 usable survey responses after excluding respondents who: 1) only answered the preliminary demographic questions (n = 44), 2) did not fish in the survey area (the Northeast U.S.; n = 7), or 3) engaged in aquaculture or party and charter boat fishing without also participating in commercial fishing (n = 21). The number of usable survey responses represents roughly 0.5% of the population of more than 49,000 commercial fishers in the Northeast region [41]. Survey respondents were from 158 zip codes in 14 states (Fig 1) and included fishers using a total of 23 gear types (S4 Table) and targeting a total of 81 species of marine and coastal fish and invertebrates. Species were collapsed into species groupings, and then into 7 fisheries types representing similarities across species, habitats, and gear types (Table 1).

**Table 1:** Description of fishery groupings based on survey responses.

Fishery	Species included
Marine benthic fish	Northeast groundfish species (cod, haddock, flounders, pollock, redfish, halibut), summer flounder, black sea bass, tautog, hake, whiting, skates, monkfish, spiny dogfish, smooth dogfish, golden tilefish, blueline tilefish
Marine small pelagics	Longfin (loligo) squid, shortfin (illex) squid, butterfish, mackerel, Atlantic herring, shrimp
Marine large pelagics/ highly migratory species	Spanish mackerel, King mackerel, dolphin/mahi, wahoo, sharks, little tunny, bonito, albacore tuna, yellowfin tuna, bluefin tuna, bigeye tuna, swordfish
Marine bivalves	Sea scallops, ocean quahogs, surf clams
Marine crustaceans	Lobster, Jonah crab, rock crab, red crab
Coastal finfish	Bluefish, striped bass, menhaden, sea trout/speckled trout, spot, croaker, drum, weakfish, toadfish, sea mullet, blowfish, American shad, baitfish, silversides, gudgeon, minnows
Coastal invertebrates	Shellfish (oysters, steamers/soft clams, sea urchins, quahogs/hard clams, razor clams, bay scallops, periwinkles), blue crabs, green crabs, conch, whelk, horseshoe crabs, starfish

**Fig 2:**
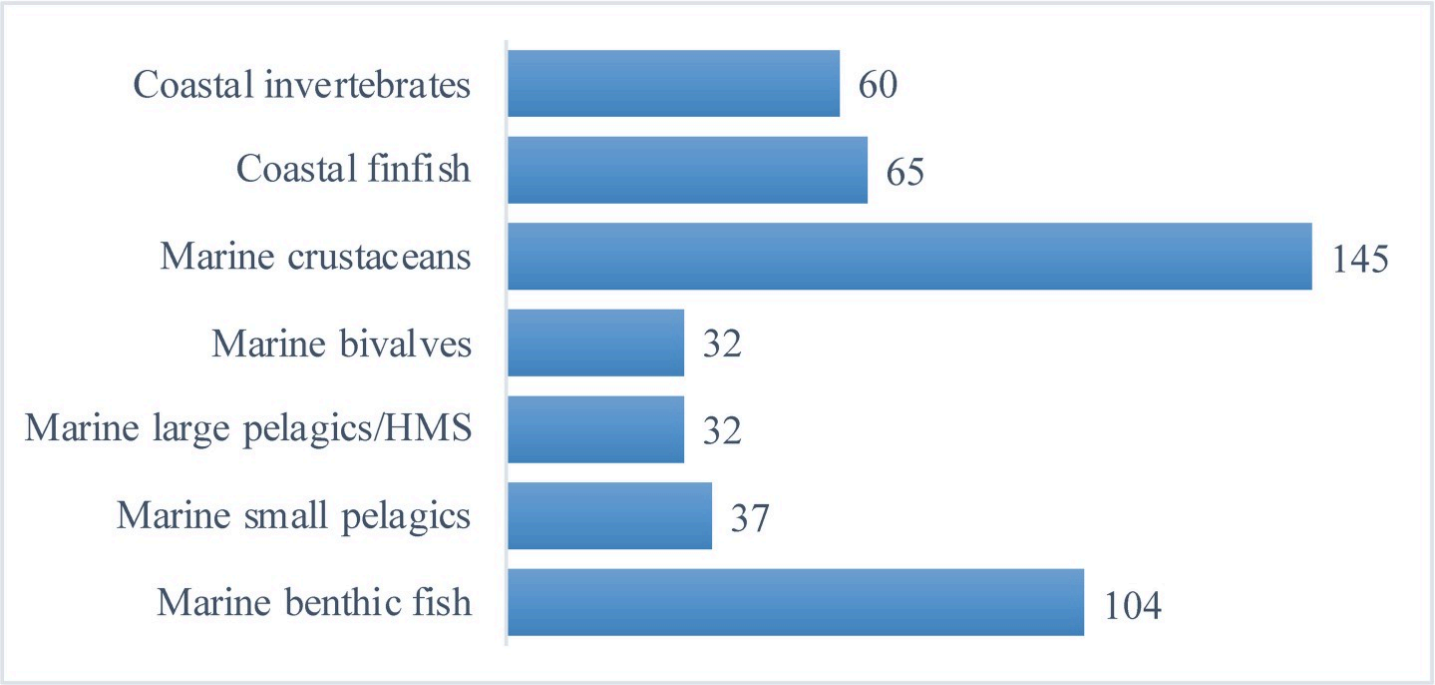
Number of survey responses by fishery. Many fishers participated in more than one fishery (and thus total responses are greater than total number of surveys).

Overall, based on a review of summary data on respondents, our survey sample was fairly representative of the population of commercial fishers in the region, with respondents participating in a broad range of fisheries from small-scale, inshore fisheries to industrial, offshore fisheries. As described above, fishers from Maine were overrepresented in the survey. Relying largely on survey distribution from industry organizations and fisheries management agencies contributed to somewhat uneven response rates, with Massachusetts fishers in particular being underrepresented. The largest percentage of respondents (56%) reported marine crustaceans (lobster and crabs) as a fishery in which they participate, followed by fisheries for various groundfish and other marine benthic finfish species (Fig 2). Lobsters are targeted by the vast majority of Maine fishers [47], although numerous lobster fishers from other states are also included in the survey. Roughly two-thirds of survey respondents reported targeting more than one species and typically participated in more than one fishery, with many listing at least five or ten species, indicating a diversity of fisheries and survey participants included.

Respondents represented a range of fishing experience, and included a large percentage of older, highly experienced fishers, representative of the demographics for fishers in the region, who tend to be older and have often been in the industry for many decades [e.g., 48]. The modal respondent had more than 35 years of industry experience (25.9% of respondents) (Table 2) and was 55–64 years of age (26.2% of respondents), with 41.9% of respondents age 55 or older. The majority of survey respondents were full-time fishers; 78.2% earned at least 75% of their income from fishing, while another 8.6% earned more than 50% of their income from fishing (Table 2). The majority of fishers were part of relatively small-scale operations, with the modal boat size reported as between 30–49 feet (9.1–14.9 meters; 51.0%), and 79.7% of vessels included in the survey (respondents could report more than one vessel size where they owned or worked on more than one vessel) under 50 feet (15 meters) in length (S4 Table). While there are several important fisheries that typically rely on larger (>50 feet), offshore vessels (e.g., scallop, squid, and some groundfish), there are numerous fisheries in the region carried out closer to shore on smaller vessels (<50 feet), including lobster, shellfish, and coastal finfish fisheries. Our survey results are representative of the diversity of fishery types that exist in the Northeast U.S.

**Table 2:** Demographic summary of survey respondents.

Role		Number of years fishing		Income earned from fishing	
Owner	58.9% (n = 152)	Less than 1	0.8% (n = 2)	Less than 25%	4.5% (n = 10)
Captain	31.4% (n = 81)	1–5 years	9.0% (n = 23)	25–49%	8.6% (n = 19)
Crew	8.1% (n = 21)	6–10 years	11.8% (n = 30)	50–74%	8.6% (n = 19)
		11–15 years	10.2% (n = 27)	75–99%	18.6% (n = 41)
		16–20 years	10.6% (n = 27)	100% of income	59.5% (n = 131)
		21–25 years	9.8% (n = 25)		
		26–30 years	11.8% (n = 30)		
		31–35 years	10.2% (n = 26)		
		More than 35 years	25.9% (n = 66)		

### 3.2 Fishery participation in the COVID-19 pandemic

#### 3.2.1. Characteristics of fishers continuing to fish vs. those not fishing

Close to sixty percent of respondents (58.6%) reported they had continued to fish during the early months of the pandemic, defined as March 2020 with the implementation of social distancing measures in their state through the date when they took the survey. A sizeable minority of respondents (41.4%) reported that they had not been fishing since social distancing restrictions went into place. We did not specifically gather data on the reasons why they had not been fishing. While the percentage of owners (58.0%) and captains (53.1%) surveyed who had continued fishing during this period was similar, the percentage of crew who had continued fishing was much higher (85.7%). This could represent the greater mobility of crew members compared with captains and owners, and their ability to find a vessel that was continuing to fish in this period, or their greater economic precarity, pushing them to continue fishing to earn income in this time while others were able to take a pause in fishing activity. Conversely, the small sample size of crew members could mean the survey did not capture crew who had already dropped out of the fishery. Respondents who fished for marine benthic fish (including summer flounder [*Paralichthys dentatus*], groundfish, black sea bass [*Centropristis striata*], and others; see Table 1) were more likely to have continued fishing during the early stages of the pandemic compared with those who participated in other fisheries (χ^2^ = 5.34, *p* = 0.02). However, there was no significant relationship between participation in other fisheries and whether respondents had continued to fish based on a Chi-square test. The relationship between the number of species a respondent pursued and whether they were likely to remain fishing was also non-significant (Independent samples t-test; *t* [242.01] = 1.09; *p* = .27; M = 2.7 for those still fishing, M = 2.5 for those not fishing). There was also no relationship between a respondent’s home state and whether or not they had continued to fish during the pandemic (χ^2^ = 18.08, *p* = 0.11).

Of those respondents who reported they were still fishing, 83.1% were full-time fishers, which we defined as earning at least 75% of their income from fishing. Among those who reported not fishing during the pandemic, 71.9%, were full-time fishers. Additionally, the modal household income was greater than $120,000 for those who had continued fishing (23.0%), compared with $61–80,000 for those who were not fishing (21.8%). However, this difference was not statistically significant (χ^2^ = 7.67, *p* = 0.26).

#### 3.2.2. Effects of the pandemic on fishers’ incomes and effort

**Fig 3:**
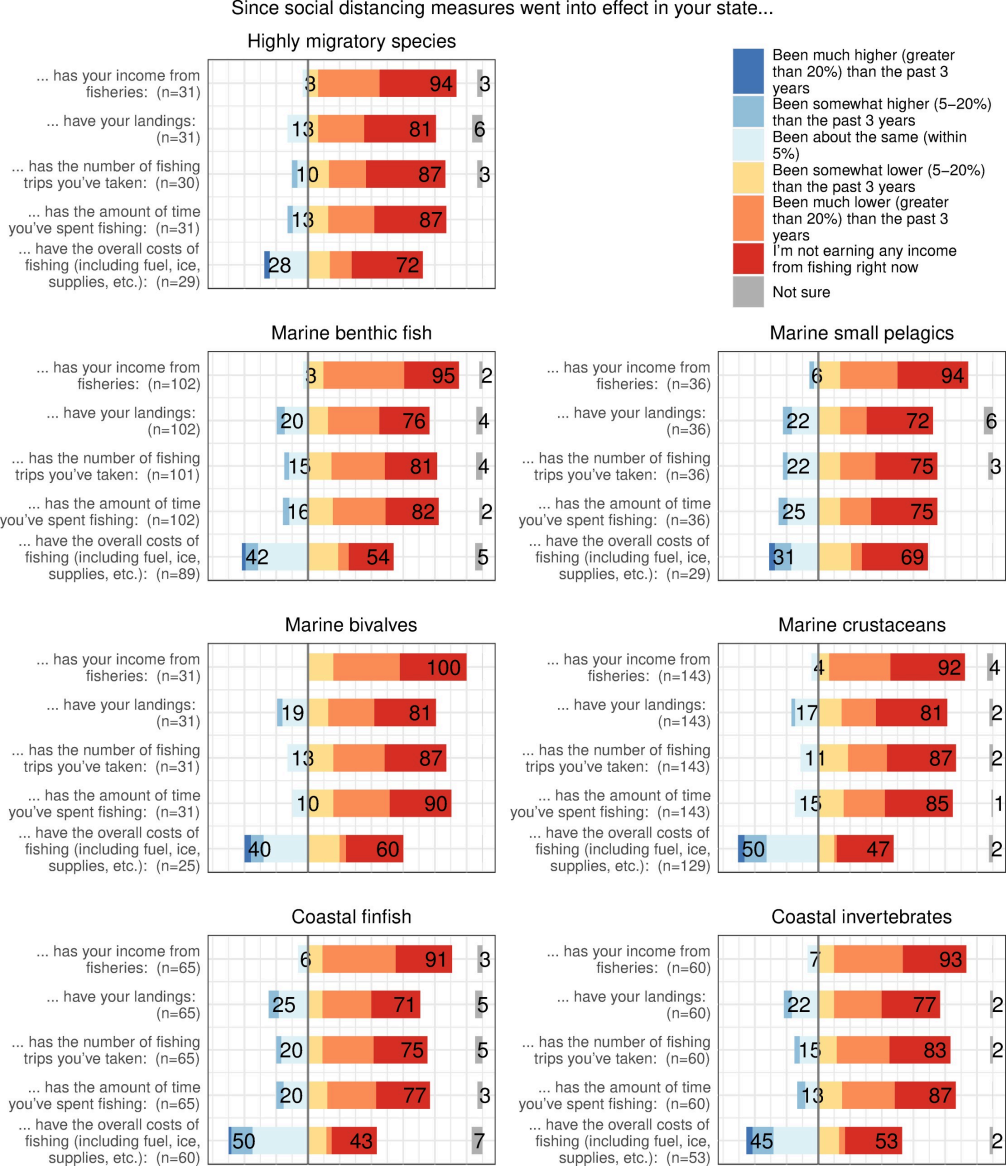
Responses to Likert-scale questions subdivided by fishery type. Responses to Likert-scale questions about changes in income, landings, effort, and cost. Overlaid numbers indicate summed percentages of responses for fishers who responded each of these categories was higher, somewhat higher, or about the same (dark blue to light blue, to the left), or somewhat lower, much lower, or they were not earning any income from fishing (yellow, orange, and red; to the right). Numbers overlaid on gray bars on the right-hand axis indicate the percentage of respondents who were unsure about the answer to the question. Note that the responses for each fishery do not sum to the total number of responses, because many respondents participated in multiple fisheries.

When asked about the effects of the pandemic on their income, 41.5% of fishers reported they were not earning any money from fishing, including some (7.8% of all fishers) who reported they had continued to fish during the early months of the pandemic. Of those fishers continuing to fish, a large majority reported a loss of income, with 62.0% of those fishers who had continued fishing reporting that their income from fishing was much lower (> 20% lower) than over the past three years, and an additional 15.3% of those who had continued fishing reporting their income was somewhat lower (5–20% lower) than over the past three years. These results held when compared across fisheries, with some differences. Fewer fishers participating in marine benthic fish and coastal finfish fisheries reported a loss of income of greater than 20%, but nearly all respondents across all fisheries reported some decline in income (Fig 3).

Trends in landings and effort as reported in the survey did not necessarily track the decline in income. A majority of those respondents who had continued to fish reported a decline in landings during the early months of the pandemic, including 64.2% of those respondents still fishing who reported their landings had been much lower (> 20%) or somewhat lower (5–20%) compared with the previous three years. However, many respondents did not report a decline in landings, with 26.5% of those respondents still fishing reporting that their landings were about the same as in the previous three years, and 4.6% of those respondents still fishing reporting that their landings were somewhat higher (5–20% higher) than in the previous three years. Fishing effort was similarly lower for a majority of respondents, but the same or higher for a subset of respondents. While 73.4% of those respondents still fishing reported the amount of time spent fishing was much lower (>20%) or somewhat lower (5–20%) than the previous three years, 20.8% of those respondents still fishing said they were fishing about the same amount as in previous years, and 3.2% said they were fishing somewhat more than over the last three years (Fig 3).

#### 3.2.3. Fishers’ confidence in the future

**Fig 4:**
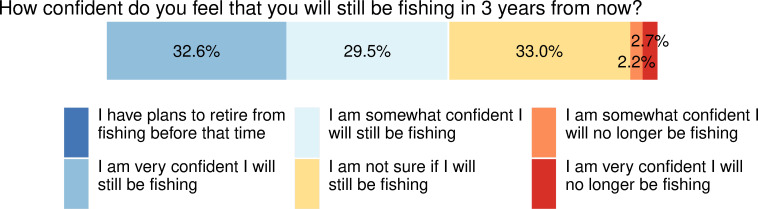
Fishers’ reported level of confidence in whether they would still be fishing in three years. Percentage of respondents who chose each response is overlaid on each bar.

When asked how confident they were that they would still be fishing in three years from the time of the survey, the majority felt confident they would continue fishing, with 32.6% responding they felt “very confident” they would still be fishing, and an additional 29.5% responding they were “somewhat confident” they would still be fishing (Fig 4). Only 2.7% were very confident they would no longer be fishing then, and 2.2% were somewhat confident they would no longer be fishing. Surprisingly, none of the respondents selected that they planned to retire from fishing within three years, particularly given the fact that more than 40% of survey respondents were over the age of 55. However, roughly one-third of respondents (33.0%) were unsure about whether or not they would still be fishing in three years. These findings suggest a considerable amount of uncertainty about the future for many fishers in the early stages of the pandemic. These results also suggest that very few who have stopped fishing during the early stages of the pandemic consider this to be a permanent departure from fishing. Even 48.6% of those who had not been fishing during the early months of the pandemic expressed they were “very confident” or “somewhat confident” they would still be fishing in three years. Overall, there was no statistical difference between those respondents who were not fishing during the pandemic and those who had continued to fish in whether they thought they would still be fishing in three years (χ^2^ = 1.91, *p* = 0.8).

### 3.3 Landings and price data

**Fig 5:**
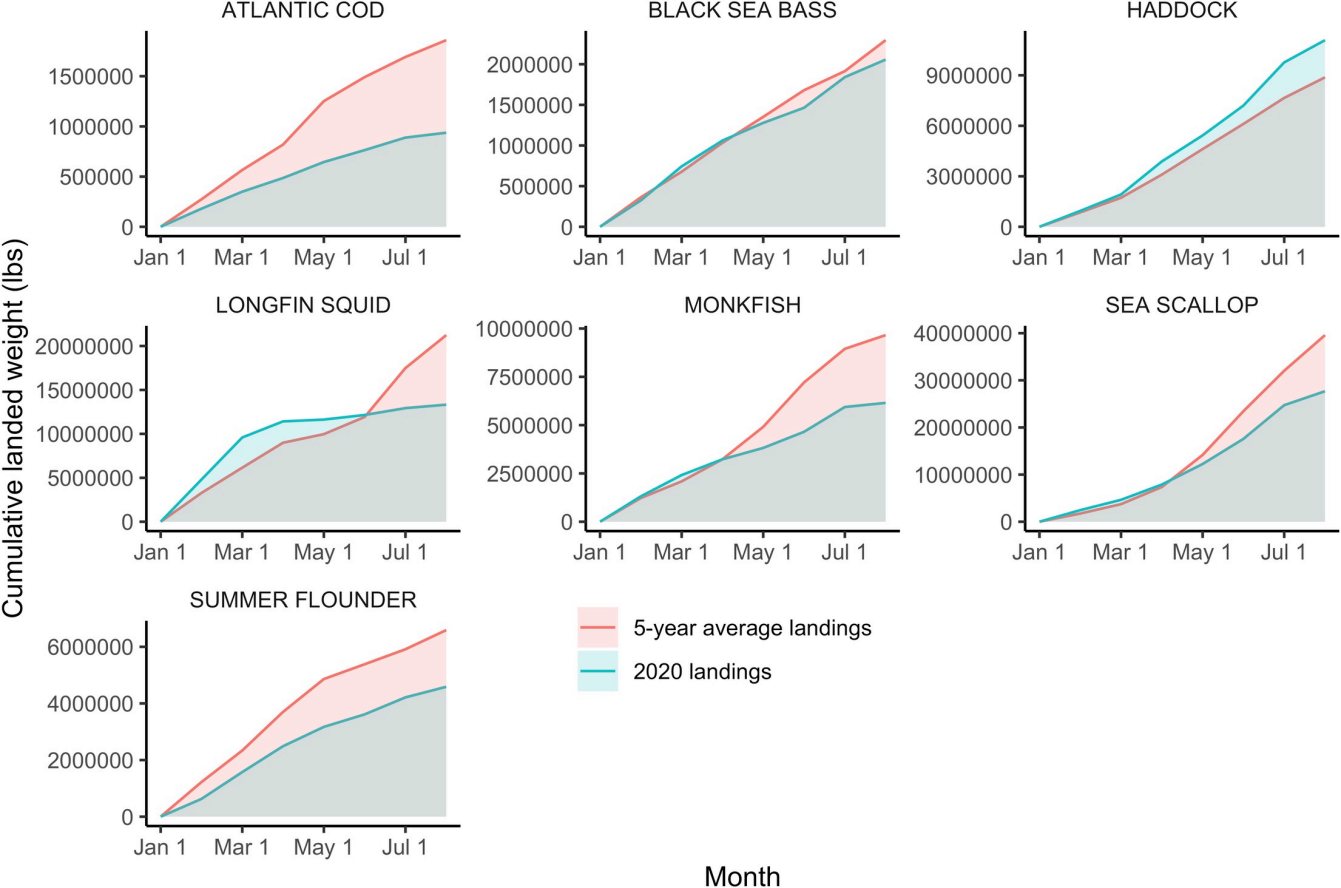
Cumulative landed weight (in pounds) of seven economically important Northeast fish species for 2020 and 2015–2019. Cumulative 2020 landings are in blue, and the cumulative average landings for 2015–2019 are in red (Data from [46]).

**Fig 6:**
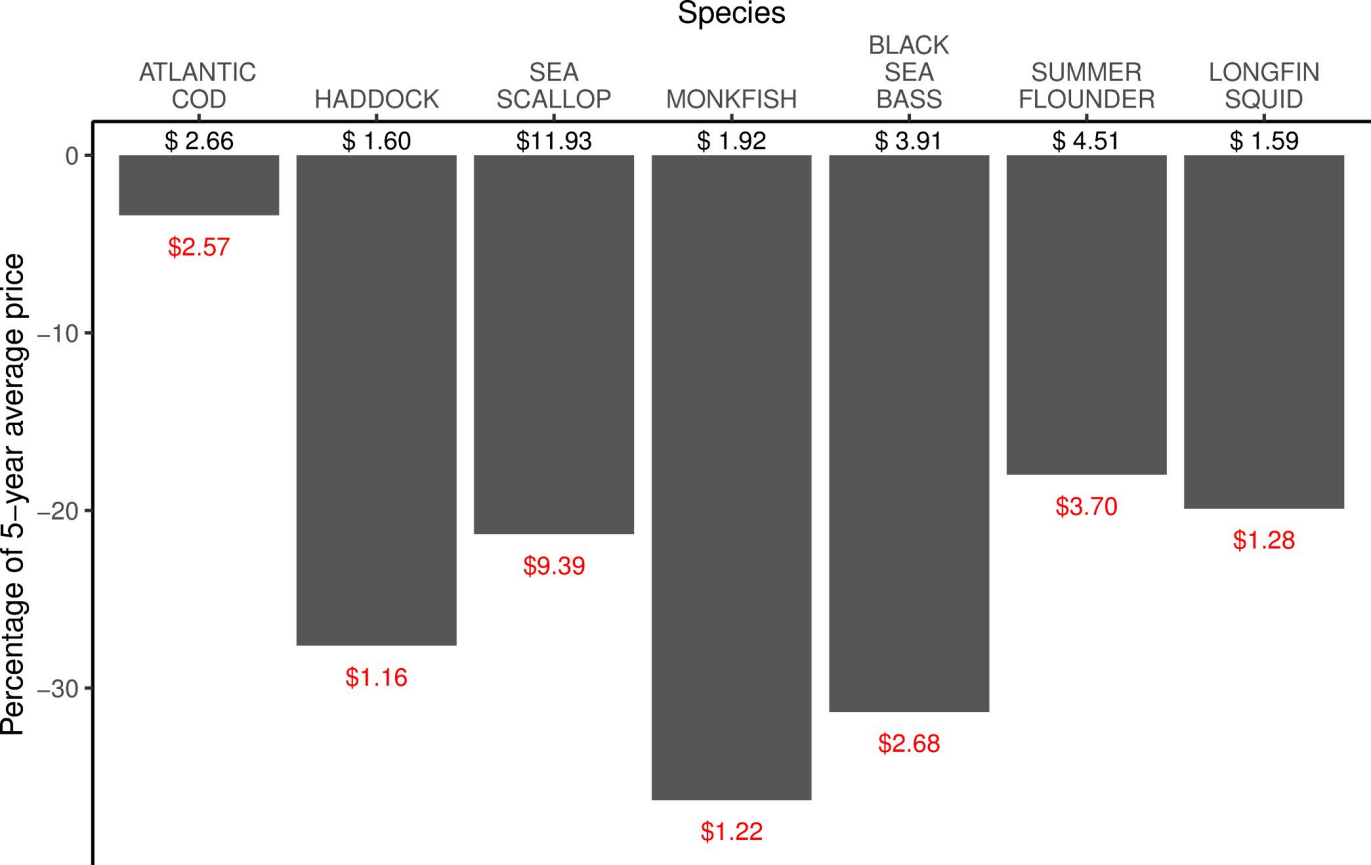
Percent difference in average price per pound of landed fish for March-June 2020 compared with 2015–2019 for seven Northeast stocks. Five-year average prices are in black, and 2020 average prices are in red (Data from [46]).

Analysis of federal fisheries landings and price data demonstrates that some fisheries experienced a large decline in landings during the early months of the pandemic, while others saw landings remain approximately on track with recent years. These trends in landings are probably largely dictated by changes in price and markets, but might also reflect changes in fisheries management between the two time periods. For example, Atlantic sea scallop landings data (Fig 5) show landings by weight that were more or less on track with or higher than the previous five years through April of 2020, while landings for the spring months (May—July) were generally lower than the average for 2015–19. Prices per pound for sea scallops, one of the highest value species in the U.S., were more than 20% below the average price for the previous five years for the first half of 2020 (Fig 6). While scallops are a seafood that many Americans are familiar with, they are often considered a luxury item and most typically eaten in restaurants or exported, often to European markets. In some cases, changes in landings could also represent some fishers shifting toward fishing in state waters (within three miles of the coast) to save on fuel costs, and either fishing for different species or not reporting them in federal landings (for fisheries outside of three miles).

**Fig 7:**
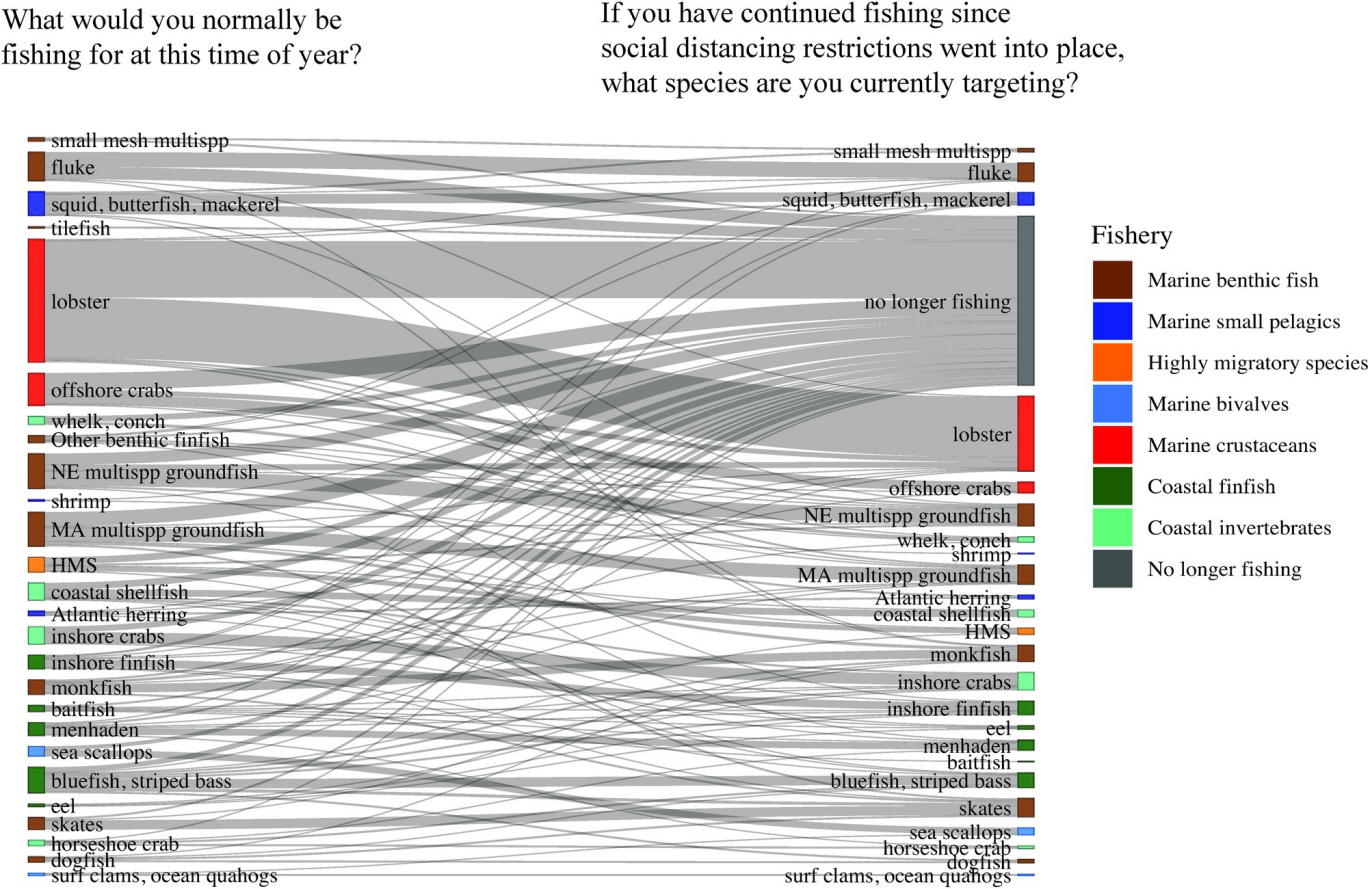
Species usually targeted in spring by respondents compared to species targeted in spring 2020. The left-hand nodes indicate the species or groups of species that respondents reported normally targeting at the time of year at which the survey was administered (May-June). The right-hand nodes indicate the species or species groups they reported currently targeting at the time of the survey. Vertical height of each node denotes the proportion of all respondents who answered species-specific questions selecting that species or species groups. Horizontal links between the same species indicate the proportion of fishers who habitually target that species and continued fishing for it during the pandemic. Horizontal links between different species indicate the proportion of fishers who switched from the species on the left to the species on the right. The “no longer fishing” node indicates the proportion of fishers who were not fishing this spring. NE = New England; MA = Mid-Atlantic; HMS = Highly Migratory Species. See Table 1 for fisheries group definitions. Note that proportions do not equal 1, as many fishers targeted multiple species. An interactive version of this figure is available as S1 Fig.

**Fig 8:**
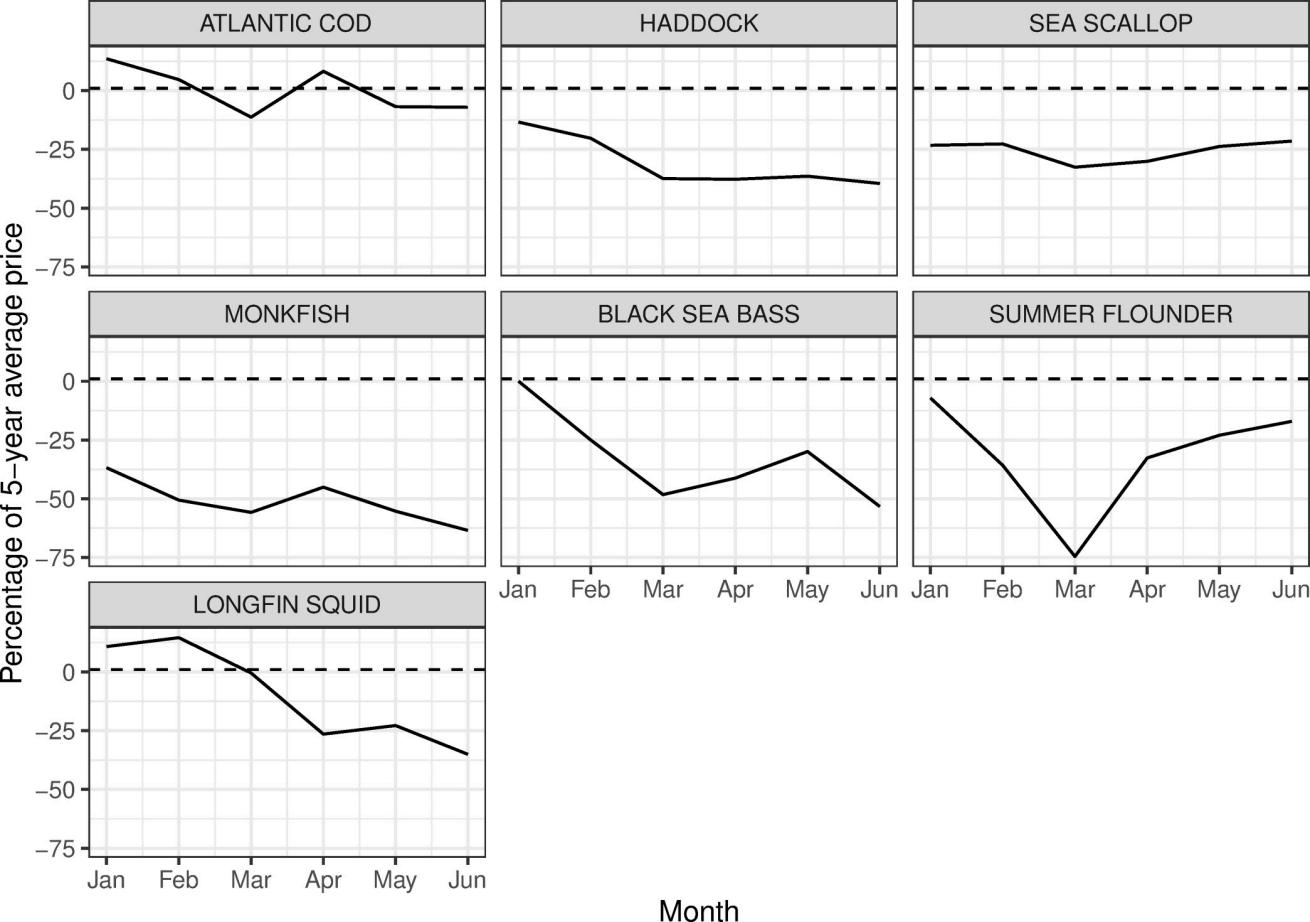
Percent difference between monthly average price for January-June 2020 and 2015–19 for seven Northeast species (Data from [46]).

Landings in the first half of 2020 for monkfish (*Lophius americanus*) and longfin squid (*Doryteuthis pealeii*) dropped to well below 2015–19 averages starting in April or May, while for both fisheries 2020 landings had started out higher than the average landings of the previous five years (Fig 5). Some survey respondents pointed to monkfish as a fishery which had lost much of its market, as monkfish is frequently exported to Asian and European markets, and as a result some fishers had switched from targeting monkfish to other species (Fig 7). Longfin squid is also often exported to European and Asian markets, or served as calamari in restaurants, with limited home consumption in the U.S. Also important to note is that many species relying heavily on export markets experienced significant declines in demand and price starting in January when the pandemic hit China, well before the pandemic began in the U.S. (Fig 8). While some species experienced some rebound in price later in the spring of 2020, prices for squid and monkfish declined through the early months of the pandemic to well below the 2015–19 average (Fig 8).

For many other fisheries, landings in the first half of 2020 more closely tracked 2015–19 averages. For example, landings of black sea bass (*C*. *striata*) were similar to the average landings of the previous five years for most of the first six months of 2020, and at times exceeded 2015–19 average landings for certain weeks. Prices for black sea bass had fallen considerably in the first half of 2020 compared with 2019 but were still relatively higher than for other species often targeted in the same fishery but not included in our analysis, including scup and tautog. Additionally, the commercial quota for black sea bass increased by 59% in May 2020, providing more opportunities to fish this stock. These factors may have supported continued effort in this fishery, along with some fishers switching from other species to targeting black sea bass.

Other species demonstrate landings and price trends that remained strong in the first half of 2020. Landings of Atlantic cod were significantly lower in the first seven months of 2020 compared with 2015–19, but prices remained steady for this relatively high value species. Landings of haddock (*Melanogrammus aeglefinus*) in the first half of 2020 were more or less on track with the 2015–19 average, but in June and July of 2020 far exceeded the average landings from the previous five years for the same time period. The reasons behind the jump in landings are complex, but likely include increased availability from a very large year class that had just recruited into the fishery, a large quota that enables fishers to target them when quotas for other species in the multispecies fishery are low, as well as $20 million in funding allocated from the USDA to purchase three species of groundfish in the Northeast including haddock, along with pollock and redfish, which likely helped to buoy the price of haddock during this time and support the fishery [49].

### 3.4 Pathways for economic impacts of the pandemic

**Fig 9:**
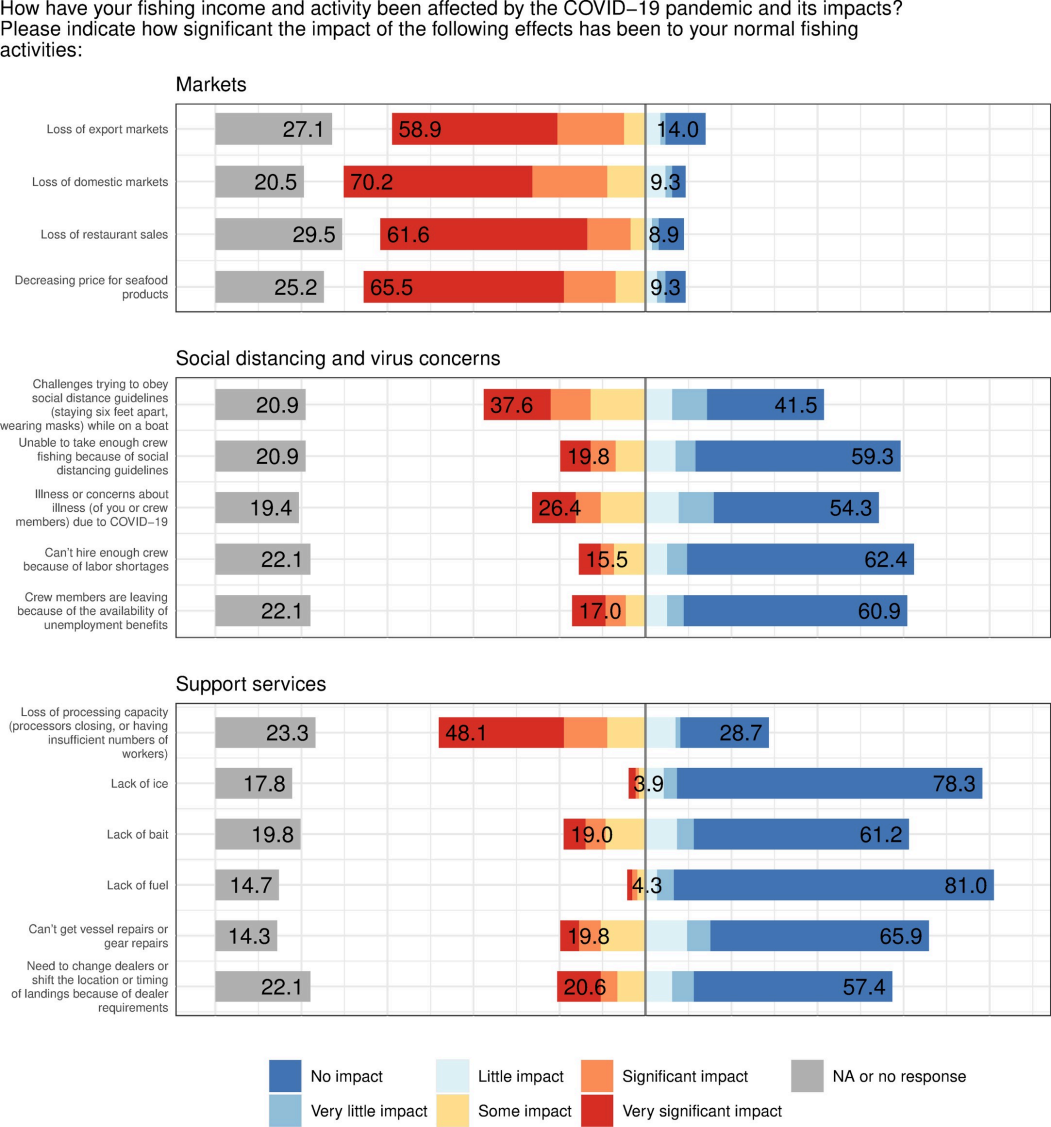
Responses to Likert-scale questions about three categories of drivers on fishers’ income and activity. Responses are grouped into three categories: markets, social distancing and concerns about illness, and availability of support services. Numbers to the left of the grey line indicate the summed percentage of fishers who selected that each driver had a very significant impact, significant impact, or some impact (red, yellow, and orange), while numbers to the right of the line indicate the summed percentage of fishers who selected little impact, very little impact, or no impact (light blue through dark blue). Numbers in gray bars are the percentage of respondents who did not answer or selected ‘N/A’.

As revealed by a series of Likert-scale questions about the impact of various drivers on respondents’ normal fishing activities, respondents’ most prevalent concern was the loss of markets for their catch. The greatest proportion (61%) of respondents cited the loss of domestic markets as having a significant or very significant impact on their fishing activity, followed by decreasing prices for seafood products (59% of respondents), loss of restaurant sales, which overlaps with loss of domestic markets (58%), and loss of export markets (54%) (Fig 9). Loss of processing capacity was also a substantial factor, with 39% of respondents labeling it as a significant or very significant impact. Only a minority (25%) of fishers reported challenges trying to obey social distancing guidelines as having a very significant or significant effect on their fishing activity. There were no significant differences in which fishers reported social distancing to be a challenge based on vessel size (χ^2^ = 2.75, *p* = 0.6). Most respondents (>80%) did not consider problems with hiring or retaining crew, concerns about illness, or issues related to shoreside support services (e.g., ice or bait) as having a significant effect on their businesses. These findings support the argument that the near-term impact to the fishing industry was largely economic, and pertaining to supply chain disruptions caused by stay-at-home measures, rather than directly related to fishers’ concerns about the virus itself.

In addition to the Likert-scale scores, respondents were given space to describe other impacts of the pandemic on their business. Here six respondents cited issues with crew, including that crew members were not interested in fishing while they could receive Paycheck Protection Program (PPP) loans or unemployment insurance, or that they were seeking employment elsewhere because of the uncertainty of fishing. Some mentioned that social distancing was not possible on a boat, and this also created issues with crew, either with retaining crew or leading to concerns about exposure to the virus from crew members. These fishers were among those who responded that social distancing was affecting their fishing ability. One reported not fishing so as to not risk getting exposed to COVID-19 from crew members, and others reported taking fewer or no crew while fishing to limit the potential for exposure.

Many respondents provided additional information about challenges in selling their catch, including in some cases processors or dealers who were either closed or setting limits on catch because their capacity was limited or they did not have sufficient freezer space. One fisher called it a “perfect storm of problems”, between the loss of exports and the loss of restaurants. Some lobster fishers specifically cited tariffs on seafood exports imposed by China before the pandemic as a compounding factor in the impacts of the pandemic, while others mentioned an increase in Canadian imports starting before the pandemic as another factor affecting the lobster industry specifically. These highlight the extent to which the pandemic may have exacerbated existing challenges fishers were already facing.

As most fisheries are of a seasonal nature, the immediate impacts of the pandemic, social distancing requirements, and the economic fallout affected different fisheries in different ways. For example, in the American lobster fishery, many respondents added that the season for lobsters in Maine, where the bulk of U.S. lobster landings are made, hadn’t really begun in earnest yet as of the time of the survey. The lobster season ramps up when the water warms and lobsters shed their shells, moving into shallower, warmer water and making them easier to catch, and corresponding with an increase in summer tourism. Indeed, some of the fishers in our survey who were not fishing at the time of the survey may in fact have been waiting for the lobster season to ramp up, although many of the lobster fishers who responded to the survey also pursued other species. Accordingly, many lobster fishers in northern New England had not yet experienced the full impacts of the pandemic on their businesses but were concerned about what the summer would bring. Likewise, many fisheries throughout the Northeast are tied to an increase in summer tourism, particularly those that rely heavily on restaurant sales rather than export markets, and these were likely to be heavily impacted by a slowdown in tourism anticipated across many tourism-dependent coastal communities.

### 3.5 Adaptations

**Fig 10:**
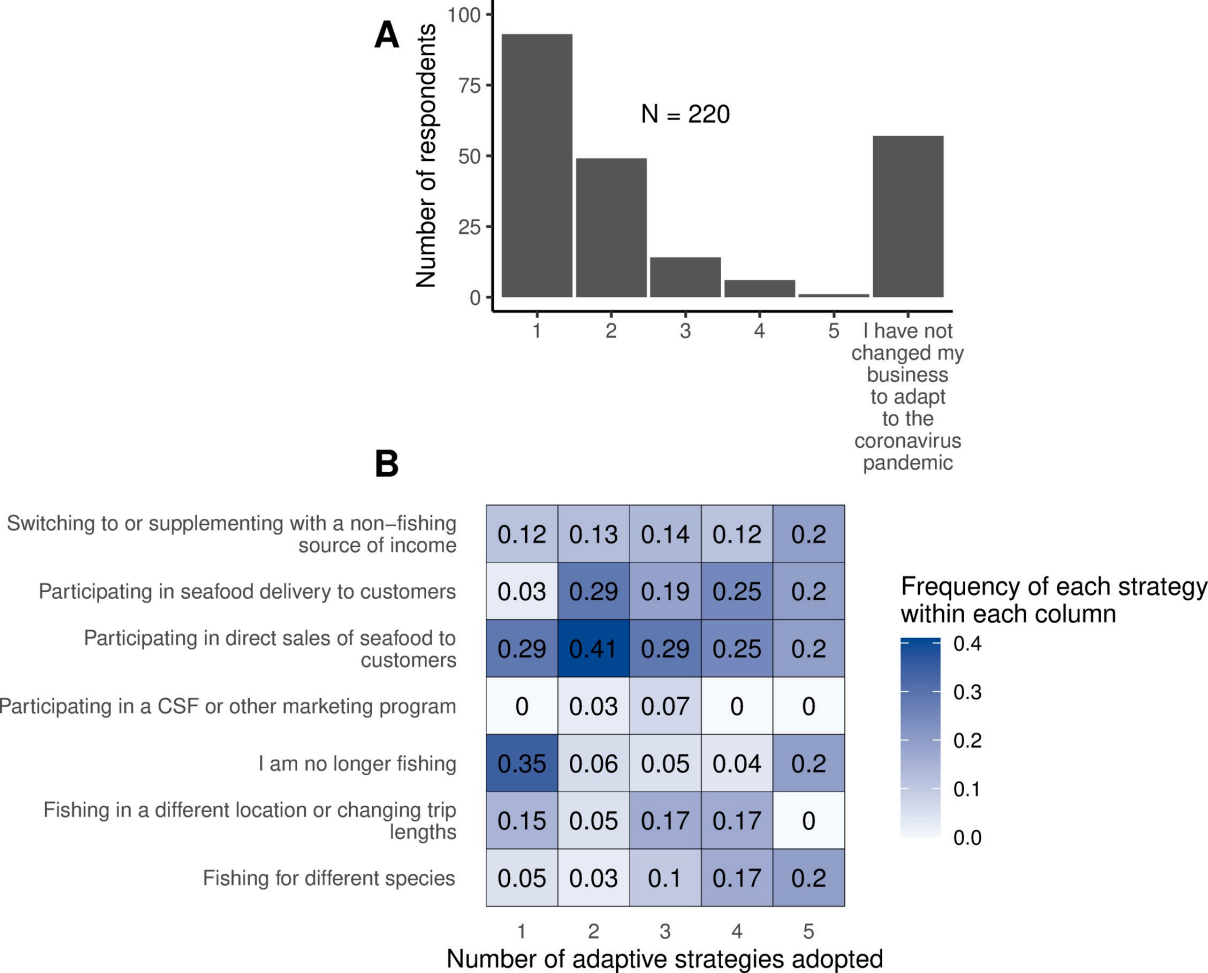
Number of adaptation strategies selected by respondents, and frequency with which each type was selected. A. Histogram showing the number of respondents who chose one or multiple adaptation strategies to adapt to the COVID-19 pandemic, including those who did not adapt their business. B. The frequency of each adaptation strategy among respondents who chose 1, 2, 3, or more adaptation strategies. Frequencies sum down the columns but not across rows.

Of the respondents who answered a question about adaptation strategies (n = 220), 42% reported deploying a single adaptation strategy, with an additional 22% using two strategies, 6% using three, and 3% deploying more than three adaptation strategies (Fig 10). About 26% of respondents stated that they had not changed their business to adapt to the coronavirus pandemic (Fig 10).

The most common adaptation strategy selected was participating in direct sales to consumers (39%), followed by seafood delivery to consumers (21%). Most of the respondents (87%) who reported participating in seafood delivery were also participating in direct sales, and presumably the seafood delivery initiatives frequently consist of delivery directly to consumers. This was consistent with reports in the media of direct sales and delivery as a strategy for a number of fishers who were unable to access their usual foreign and domestic markets [21]. While fishers targeting some species, notably crabs and lobsters, have always been able to sell their catch directly, the pandemic afforded new opportunities for direct sales such as in Rhode Island, where state fisheries managers revised regulations to enable direct sales of finfish [20], and in many other states where state- or community-level initiatives sought to promote direct sales of local seafood.

A number of respondents also chose “Fishing in a different location or changing trip length” as an adaptation (10.8%). In the free response section where they were asked to describe further, a few respondents described fishing on shorter trips or closer to shore, while others described longer or farther trips in pursuit of different species. “Fishing for different species” was also reported by several respondents (6.5%), who may have switched to more marketable species, or stopped targeting certain species for which the market wasn’t holding up. Some survey respondents reported changing the species they were targeting after the start of the pandemic compared with what they would normally be targeting during the same season (Fig 7). For example, one respondent who had reported typically fishing for monkfish and little and winter skates (*Leucoraja erinacea*, *Leucoraja ocellata*) with a gillnet, and would traditionally be targeting higher-value monkfish at that time of year, had switched to skate because there was no market for monkfish. Fishers reported targeting significantly fewer species in 2020 (M = 1.7) than they would ordinarily be targeting (M = 2.0) during the same season (May-June), (paired samples t-test, *t* [279.99] = 2.01; *p* = .04) (Fig 7).

A large proportion of respondents reported relying on or anticipating financial support from the government to help them address short-term or future lost income. Of total respondents, 60.0% said they had received or anticipated receiving some form of government support. Of these, 61% were receiving one source of support, 21% were receiving two, and 12% were receiving or anticipating to receive support from three or more sources. The most commonly reported source of support was the Economic Impact Payment checks (commonly referred to as stimulus checks) that the federal government sent out to all U.S. taxpayers below a certain income threshold, with just over half of those respondents receiving federal assistance (62%) reporting they had received or expected to receive a stimulus check. Additionally, 41% of those respondents who had received or anticipated receiving government support listed the Paycheck Protection Program (PPP) loan, which allows small businesses to receive a loan to continue to pay their employees. However, most often crew members on fishing boats are considered to be self-employed; the Small Business Administration rules governing the eligibility for PPP loans originally excluded self-employed fishers from protection, but this rule was amended in late June 2020 (after the survey had closed) to include fishers [50]. While some of the respondents who listed the PPP as a source of support may have been eligible, others may have been anticipating their eligibility but were not able to access this funding at the time of the survey. Similarly, 8% of respondents listed fishery disaster assistance from NOAA (allocated through the CARES Act) as an existing or future source of assistance, but at the time of the survey, this disaster assistance was not yet available, as individual states had yet to make decisions about how to allocate funding.

The prevalence of financial support from the government points to the use of government support as a short-term adaptation strategy for the fishing industry to bridge a gap in income. However, we found no relationship between whether respondents stated they had received government support and whether they were still fishing (χ^2^ = 0.007, p = 0.9). Fishers who were still fishing were receiving similar numbers of types of government support as those who had stopped fishing, although they differed in the types of support, with those fishers not fishing more likely to be receiving pandemic unemployment assistance, and those still fishing more likely to anticipate receiving fishery disaster assistance from the CARES Act (funds for which were not yet available at the time of writing) (S2 Fig).

## 4. Discussion

This study found that the early stages of the COVID-19 pandemic caused significant disruptions for the commercial fishing industry in the Northeast United States. While there were some differences among fisheries and among fishers with different demographic traits, the near-term economic impacts of the pandemic were nearly ubiquitous throughout the industry and deeply felt. At the same time, the industry demonstrated elements of resilience that are likely critical in allowing the industry to weather a very difficult period of time, including making near-term adaptations to fishing and marketing strategies. Here we also discuss the decision of many fishers to continue fishing during the pandemic, and whether this is suggestive of a resilient fishery or simply a lack of alternatives.

More than 40% of fishers reported at the time of the survey that they had not been fishing since social distancing restrictions went into place in mid-March, 2020. This in many cases means the loss of at least two to three months of fishing income during the early stages of the pandemic. However, some of these fishers may not have been fishing for reasons other than those directly attributable to the pandemic, including those participating in seasonal fisheries that had not yet begun, or those not fishing for other reasons (e.g., boat repairs). Nearly all fishers surveyed reported a loss of income, with well over 80% reporting that they had either seen a significant decline in their income from fishing, or they were not earning any income from fishing at the time of the survey.

The drivers behind these economic impacts are numerous and demonstrate the complexity of the economic and social consequences of the COVID-19 pandemic. However, those drivers related to impacts on the seafood supply chain, including disruptions of both domestic and export markets, and a concurrent drop in price for many seafood products, appear to have had the most significant effects on fishers’ livelihoods in the short-term according to survey findings. Many of the disruptions experienced by fishers during this pandemic were in fact disruptions of the seafood supply chain [1], such as a loss of export markets or a lack of domestic markets other than restaurants. Promoting resilience and stability of the seafood supply chain in the long run through, for example, expanding the demand for domestic products and expanding retail markets, may be just as important to fishers’ livelihoods as maintaining their ability to fish [51].

When comparing survey respondents who had not been fishing since the start of the pandemic to those who had continued fishing, few trends uncovered in the data explain the differences in participation among respondents. There were no differences found in whether respondents were still fishing by state, whether or not they were receiving financial assistance from the government, or how many species the fishers generally targeted (i.e., how diversified they tend to be). These findings suggest a likelihood that numerous individual-level factors, such as the availability of alternatives, levels of job satisfaction, or one’s own perceptions of risk, may determine whether fishers make the decision to continue fishing [52].

In one exception, of the fishery types included in the survey, those fishers participating in fisheries for marine benthic species, which includes groundfish, monkfish, skates, dogfish, black sea bass, and a handful of other species, were more likely to have continued fishing during the early stages of the pandemic than fishers engaged in other fisheries. There are likely multiple explanations for this finding. One may be that these fishers tend to be more diversified and may target multiple groundfish species with the same gear, including otter trawls and gillnets, allowing them to switch among species and target those for which the markets have held up better. Another explanation may be that these fisheries target whitefish species for which retail demand may have held up better than for some more luxury species such as lobster and scallops (i.e., they are not as reliant on restaurant sales and may be more appealing to home cooks), and they may not rely on export markets to the same extent as some other fisheries. These findings suggest fishers participating in marine benthic fisheries may in fact be more resilient than those in other fisheries, perhaps as a result of the multi-species nature of these fisheries.

### 4.1 Adapting to near-term change

It has been well documented that many fish stocks experienced a respite from fishing pressure, and subsequent population recovery, during World War II [53], leading some to point to the pandemic as an opportunity for a slowdown in fishing pressure that might benefit fish stocks akin to a fishing moratorium [3]. Given the survey findings paired with preliminary landings data for several fish stocks in the region, this effect is not likely to be observed for fish stocks in the Northeast U.S. based on trends in commercial fisheries in the first half of 2020. While a modest release in fishing pressure may have been experienced in the short-term for some stocks, for others no such reduction in fishing pressure was apparent during the early stages of the pandemic.

Many of those fishers who were still fishing reported that they were fishing less often and landing fewer fish than they would ordinarily be at this time of year. However, landings data for a number of fisheries tell a somewhat more complicated story. While landings for some species were down considerably from the same time period in recent years, landings for others were on track with those from recent years. The example of haddock, for which landings were higher than in previous years, highlights just how complex fishers’ responses to the pandemic are, and how much they are mitigated by ecological, economic, social, and management factors.

While price and markets are important drivers of landings, other factors related to the specific fisheries management regime for each species, including quotas, catch limits, and seasons, must also be considered when comparing landings, and may in part dictate the extent to which certain species were targeted during the early months of the pandemic. For example, the annual catch limit (ACL) for sea scallops in 2020 was about 20% lower compared with 2019. Additionally, the fishing season for sea scallops began April 1, so many fishers may have been waiting to fish until later in the season, hoping prices would rebound. Each of these factors could also help to explain lower sea scallop landings compared with previous years. Additionally, black sea bass and summer flounder are managed at both the federal and state levels, and the states further divide quota by seasons. Some fishers may have felt compelled to target these stocks as they came upon the end of a season rather than forgo the opportunity to harvest this quota, whereas for species for which the quota is allocated annually, such as sea scallops, fishers have greater flexibility in when to use their quota and may prefer to hold off and await higher prices. Thus the level of flexibility granted to fishers by the management regime for each fishery may also affect fishers’ behaviors during the pandemic.

Despite declines in prices, losses of markets, processing bottlenecks, and the challenges of maintaining social distancing while fishing, the majority of fishers who responded to the survey had continued fishing through the early months of the pandemic. Unfortunately, despite continuing to fish, the low prices and lack of markets may mean fishing is only marginally profitable if at all. Survey data demonstrated that most of those fishers who had continued to fish had experienced a significant decline in income. Data collected from the survey support some anecdotal reports that many fishers continued fishing in order to earn enough money to pay bills and to maintain their crew, and may have been fishing at a loss or breaking even in hopes circumstances would improve in the future.

Continuing to fish despite low economic returns may serve a few purposes. It may permit fishers to maintain their identity as fishers, which is important for many participants in the fishing industry and may be even more so under times of stress [54]. It allows them to maintain their fishing history and annual quotas, which in some cases are used to determine future permit eligibility or allocations. Continuing to fish potentially enables them to earn enough to continue payments on vessels for those fishers who own vessels, and to pay their crew and retain good crew members. At the community level, maintaining fishing activity also helps to maintain the infrastructure of fishing communities, including docks, processing facilities, gear storage, etc., which is important because these types of infrastructure are highly vulnerable to being lost to gentrification [55]. Additionally, fisheries markets can be easily lost to substitutes including cheaper exports when one species is not available [56]. Maintaining supply chains may ensure these markets are not lost to foreign imports or other similar species. In sum, fishing through a disturbance such as the COVID-19 pandemic may be an indicator of resilience, in that these fishers are maintaining the structure and function of the fishing industry.

### 4.2 Mechanisms of resilience

Fishers are accustomed to short- and long-term uncertainty, including uncertainty related to both biological and economic conditions [57, 58]. While there are few modern examples of such wide-scale disruption as the COVID-19 pandemic to look to as analogues to understand the lessons and outcomes of this particular event, the literature contains numerous examples of how fishers respond to other sorts of natural and economic challenges [e.g., 59–61].

Many short-term coping strategies have been documented that allow fishers to ‘ride out the storm’ of poor ecological or economic conditions, which may include seeking additional or alternative employment in other sectors, depending on support payments from the government, or shifting some economic responsibility to other members of the household [62]. Each of these strategies is seen in the results of our survey, including relying on the income of a family member who is still employed, engaging in additional non-fishing activities for income (survey respondents included landscaping, carpentry, real estate, and other marine trades such as vessel surveyor, among many others in their responses), and relying on government support (including short-term support such as unemployment assistance and stimulus checks, as well as long-term support including social security payments). Longer-term adaptation responses to stresses in marine social-ecological systems may include individual or community restructuring toward alternative employment, or shifting fishing activities toward different species or using different gear types [62]. The ability of fishers to engage in longer-term adaptations will also depend on the availability of alternative employment opportunities, as well as the availability of opportunities to move into new fisheries, including the availability of permits or quota.

Among the adaptation strategies named in the survey, many fishers reported participating in some sort of alternative marketing or distribution program, frequently by selling seafood direct to consumers either from their vessel or through residential delivery programs. Many of these initiatives began or were significantly accelerated at the start of the pandemic as fishers looked to alternative distribution for their products. Direct sales initiatives allow fishers to shorten the supply chain and to receive more of the value, and often a higher premium, for their catch, while enabling consumers to support local food systems [63]. Particularly as elements of the seafood supply chain failed, including processors being shut down because of COVID-19 outbreaks [17], or export markets being halted, these alternative marketing strategies may be allowing some fishers to weather the early stages of the pandemic. The trend in direct-to-consumer sales may represent a long-term adaptation strategy, particularly as the pandemic lingers and as restaurant dining sees a long-term decline [1] or suppliers derive other non-monetary value from participation [64]. On the other hand, the volume of fish sold through direct sales is typically a fraction of what fishers need to sell to turn a profit, so this alone is unlikely to be a sufficient adaptation strategy for many fishers who have seen a significant drop in income.

Diversification can be a critical component of adaptive capacity for fishers. Fishers have historically relied upon portfolio diversification (holding the necessary permits, quota, or gear to target multiple species), and switching among fisheries and species, to respond to changing ecological, economic, or management conditions [65, 66]. Survey results found some fishers who reported switching among species, and the mean number of species fishers reported targeting during the early stages of the pandemic had declined somewhat from pre-pandemic conditions, indicating those who are capable of switching among species may have stopped targeting species that are less profitable or where demand had shrunk significantly. Where fishers were more strongly impacted by the loss of export or domestic markets as a result of the pandemic, switching among species within their portfolio may enable them to target species which either had a market that had held up better, or had better opportunities for long-term storage, including freezing. Flexibility can be an important component of resilience [6]. However, to the extent the impacts were driven by fishers’ inability to go fishing because of social distancing requirements or a lack of support services, diversification within fisheries does not increase adaptive capacity.

The decision to stop fishing temporarily or altogether in pursuit of other employment or retirement can also be considered an adaptation strategy. Indeed, those survey respondents who had not been fishing since the start of the pandemic may be considered to be pursuing an adaptive strategy. Some authors have pointed to exiting the fishery and pursuing other work or income sources as a resilience strategy for fishers [6, 23, 67]. In a study of Australian fishers, Marshall and Marshall [6] found that while in some cases fishers demonstrating high resilience may remain in a fishery after a significant institutional shift because they are able to manage the risks resulting from institutional change or they have the ability to adapt to change, those fishers who leave the industry after such a change may also be demonstrating social resilience, because they have the ability to transition to a different livelihood.

Indeed, there may be a significant number of factors that determine whether or not fishers decide to stop or to continue fishing [68], especially when faced with circumstances such as the COVID-19 pandemic. In one study of Maine fishers asked about their perceptions of resilience, many of the fishers surveyed described resilience as “survival” or continuing to be in the fishery despite the numerous challenges they face [69]. Some studies have found that fishers are often reluctant to exit a fishery, even when it is the economically rational decision, sometimes because of the non-economic benefits, including job satisfaction they may derive from fishing as a livelihood [54, 70]. In other cases, fishers who choose to persist in a fishery may be doing so because of a lack of other livelihood options, or because of the capital they have already invested in the fishery, rather than as an indicator of their resilience [68]. Results of this research did not find many clear factors that could predict whether or not fishers were likely to continue fishing, highlighting the complexity of this decision and the number of factors that come into play for fishers. Nevertheless, more detailed surveys focusing on particular groups of fishers including analyzing some of the factors described above may help to shed light on individual factors that could predict whether fishers are likely to continue fishing or exit the fishery.

In considering the 41% of fishers who reported they had not been fishing since the start of the COVID-19 pandemic in the U.S., it remains to be seen which of these fishers had entirely exited the fishery and will choose not to return to fishing as a livelihood in the future. However, as few of the fishers surveyed expressed confidence that they would no longer be fishing three years into the future, it would suggest very few had made a decision to exit the fishery permanently at the time of the survey. Thus they may have been coping with the pandemic in the short-term by keeping their boats tied to the dock rather than incur the costs of going out fishing, awaiting a return to higher prices, or perhaps relying on government payments or additional non-fishing sources of income, rather than making a long-term adaptation to the pandemic by seeking an alternative source of livelihood. On the other hand, as the effects of the pandemic and its economic impacts linger into the future, and if the demand and price for seafood remain depressed, some fishers may have no choice but to exit the fishery permanently.

### 4.3 Long-term implications

This study is focused on impacts to the fishing industry during the early stages of the pandemic in the Northeast United States, when stay-at-home orders were in place, which had serious repercussions on markets, supply chains, and the ability of fishers to go fishing. As stay-at-home orders were lifted in each of these states, the immediate challenges of some of these impacts may have lessened. For example, restaurants were allowed to open, supply chain disruptions became less frequent, and concerns about maintaining social distancing aboard fishing vessels may have relaxed. Conversely, the medium- to long-term impacts of the pandemic on the industry were still very much uncertain at the time of this research. As summer arrived in the Northeast United States, the high season for tourism in many coastal communities was anticipated to be significantly curtailed by the impacts of the pandemic. This could exponentially compound challenges for the fishing industry, as a number of fisheries rely heavily on tourists and restaurant sales during this busy season, particularly with a diminished export market.

The system perturbations resulting from the COVID-19 pandemic undoubtedly increased short-term and perhaps long-term uncertainty for the fishing industry. This uncertainty, however, is also paired with ongoing uncertainty for many fisheries in the Northeast United States, a number of which have experienced recent shocks. For example, the American lobster fishery is currently subject to ongoing disputes about its role in the demise of the North Atlantic right whale (*Eubalaena glacialis*), and what restrictions should be placed on the industry to ensure the survival of this critically endangered whale species [71]. The impacts to the industry of the pandemic and its fallout may be compounded by any restrictions to gear or effort that may be placed on the industry as a result of ongoing lawsuits. A number of updated stock assessments indicate that many key stocks remain overfished, including Atlantic cod and yellowtail flounder (*Pleuronectes ferruginea*), and fisheries managers have had limited success in rebuilding their biomass [72], further reducing profitability and flexibility for many fishers. Furthermore, each of these fisheries is being affected by climate change as warming waters and other climate-driven impacts alter the distribution, abundance, and timing of numerous species upon which the commercial fishing industry of the Northeast U.S. depends [73–75]. While the commercial fishing industry as a whole in the Northeast region, and the individual fishers within the industry, have frequently demonstrated resilience in the face of change and uncertainty, the cumulative impacts of each of these stressors will pose monumental challenges.

The sudden severe shock of the COVID-19 pandemic was unforeseen and cannot be fully controlled through fisheries management measures. However, fisheries management systems can attempt to mitigate some of the effects of both short- and long-term change on fisheries social-ecological systems. Pointedly, climate change is affecting commercial fisheries globally, having repercussions for both the marine ecosystem and the fishing communities that engage with this ecosystem. Managing fisheries in a way that can increase flexibility and adaptive capacity to enable resilience will allow fisheries systems to better weather the impact of future perturbations including economic shocks such as the pandemic, and climate-driven shocks. The COVID-19 pandemic may provide a window of opportunity for fisheries managers and stakeholders to address the likelihood of future system shocks, and to learn from the current situation to build resilience for the fishery [1, 76].

While the ultimate goal of fisheries management in the United States as defined by the Magnuson-Stevens Fishery Conservation and Management Act may be to promote optimum yield to increase the long-term economic and social benefits of fisheries to the nation [77], those fishers who remain fishing may instead be reducing their effort only slightly in exchange for very low economic returns. While this strategy may confer long-term resilience to some fishers, in the short-term fishers have experienced severe economic consequences of the pandemic without conversely providing ecological benefits through a respite in fishing pressure. On the other hand, by demonstrating resilience in the face of a significant perturbation and continuing to fish, fishers may be ‘weathering the storm’ and allowing the fishing industry to survive in order to ensure these long-term benefits in the future.

## 5. Conclusions

The findings of this research indicate that commercial fishers in the Northeast United States have been significantly impacted by the early stages of the COVID-19 pandemic in numerous ways. Many fishers were coping with the challenging circumstances created by the pandemic and trying to survive through the upheaval of the fishery system by continuing to fish, perhaps less frequently, and often for much lower revenue, possibly in order to continue to make payments on boats and to their crews. Another cohort of fishers stopped fishing during the early stages of the pandemic, or perhaps did not begin their typical fishing season, as an adaptation to low prices, a lack of demand, and social distancing requirements. Survey responses suggest, however, that these fishers mostly planned to return to fishing once circumstances improved. Additionally, fishers engaged in a number of adaptation strategies from the beginning of the pandemic, including direct sales and delivery of seafood products to customers, changing their targeted species to fish those with higher demand or better prices, and frequently by changing the timing and location of their fishing trips. Many others were using federal financial assistance to meet revenue shortfalls and sustain their fishing businesses.

Many of these adaptation strategies will help commercial fishers to sustain their livelihood in the face of the monumental challenges created by the COVID-19 pandemic. However, it remains to be seen whether various sectors of the commercial fishing industry will be sufficiently resilient to rebound from this latest threat to their viability, or whether the impacts of the pandemic compounded with other impacts such as climate change, fluctuating fish stocks, and increasingly stringent regulations will significantly reshape some sectors of the industry. The findings presented in this paper reflect only the early months of the pandemic in the Northeast U.S. including the time of the initial stay-at-home orders and the early reopening of most states. While those months were a very uncertain time for the commercial fishing industry, this study by no means captures all of the anticipated impacts to commercial fisheries. COVID-19 is likely to wreak havoc on the economy and on fishers’ livelihoods well beyond the timeline of this research. The economic impacts of this pandemic on the fishing industry, as well as the disruptions to seafood supply chains, fisheries exports, local demand, and fishers’ ability to go fishing, will undoubtedly reshape the landscape of the commercial fishing industry in the Northeast U.S. for quite some time. What remains to be seen is whether these changes are short-term perturbations and responses to changing circumstances, or longer-term adaptations that may cause permanent shifts to the nature of fisheries in the Northeast U.S.

## Supporting information

S1 File Copy of Qualtrics survey disseminated to Northeast commercial fishers. (PDF)

S1 Fig Species usually targeted in spring by respondents compared to species targeted in spring 2020 –interactive version of Fig 7. The left-hand nodes indicate the species or groups of species that respondents reported normally targeting at the time of year at which the survey was administered (May-June). The right-hand nodes indicate the species or species groups they reported currently targeting at the time of the survey. Vertical height of each node denotes the proportion of all respondents who answered species-specific questions selecting that species or species groups. Horizontal links between the same species indicate the proportion of fishers who habitually target that species and continued fishing for it during the pandemic. Horizontal links between different species indicate the proportion of fishers who switched from the species on the left to the species on the right. The “no longer fishing” node indicates the proportion of fishers who stopped fishing entirely this spring. NE = New England; MA = Mid-Atlantic; HMS = Highly Migratory Species. See Table 1 for fisheries group definitions. Note that proportions do not equal 1, as many fishers targeted multiple species.
(HTML)

S2 Fig Types of government support received or anticipated to be received by fishers who are still fishing and who are no longer fishing. (PNG)

S1 Table Stay-at-home orders by state. (PDF)

S2 Table Survey responses by gender. (DOCX)

S3 Table Survey responses by race/ethnicity. (DOCX)

S4 Table Fishery characteristics, including vessel size and income, by fishery type. (DOCX)

## References

[1] Love DC, Allison EH, Asche F, Belton B, Cottrell RS, Froehlich HE, et al Emerging COVID-19 impacts, responses, and lessons for building resilience in the seafood system. SocArXiv [Preprint]. 2020 June 27, 1–22 [Cited 2020 July 1]. 10.31235/osf.io/x8aew PMC8417121 34513582

[2] Teh LCL, Sumaila UR. Contribution of marine fisheries to worldwide employment. Fish and Fisheries. 2011; 14(1): 77–88. 10.1111/j.1467-2979.2011.00450.x

[3] Bennett NJ, Finkbeiner EM, Ban NC, Belhabib D, Jupiter SD, Kittinger JN, et al The COVID-19 Pandemic, Small-Scale Fisheries and Coastal Fishing Communities. Coastal Management. 2020 10.1080/08920753.2020.1766937

[4] Folke C, Hahn T, Olsson P, Norberg J. Adaptive governance of social-ecological systems. Annu. Rev. Environ. Resour. 2005; 30: 441–73. 10.1146/annurev.energy.30.050504.144511

[5] IPCC. Climate Change 2007: Impacts, Adaptation and Vulnerability. Contribution of Working Group II to the Fourth Assessment Report of the Intergovernmental Panel on Climate Change. Parry ML, Canziani OF, Palutikof JP, van der Linden PJ, Hanson CE, Eds. Cambridge University Press, Cambridge, UK; 2007. 976pp.

[6] Marshall NA, Marshall PA. Conceptualizing and operationalizing social resilience within commercial fisheries in Northern Australia. Ecology and Society, 12(1): 1 2007 10.5751/ES-01940-120101

[7] Seara T, Clay PM, Colburn LL. Perceived adaptive capacity and natural disasters: A fisheries case study. Global Environmental Change. 2016; 38, 49–57. 10.1016/j.gloenvcha.2016.01.006

[8] White ER, Froehlich HE, Gephart JA, Cottrell RS, Branch TA, Baum JK. Early Effects of COVID-19 Interventions on US Fisheries and Seafood. OSF Preprints. 2020 June 2. 10.31219/osf.io/9bxnh PMC7753393 33362433

[9] Béné C. Resilience of local food systems and links to food security–A review of some important concepts in the context of COVID-19 and other shocks. Food Security. 2020 10.1007/s12571-020-01076-1 PMC7351643 32837646

[10] White C. China unlikely to consider lowering of lobster tariffs, despite Trump’s threat. Seafood Source. 2020 June 12. From: https://www.seafoodsource.com/news/supply-trade/china-refuses-to-consider-lowering-of-lobster-tariffs-despite-trump-s-threat?utm_source=marketo&utm_medium=%E2%80%A6

[11] FAO. 2020 Food Outlook—Biannual Report on Global Food Markets: June 2020. Food Outlook, 1. Rome. 10.4060/ca9509e

[12] Goldthwait J. State of Maine: Lobstermen are feeling the pinch. Mount Desert Islander. 2020 May 20 [Cited 2020 July 2]. From: https://www.mdislander.com/opinions/state-of-maine-lobstermen-are-feeling-the-pinch 10.1097/BPO.0000000000001610 32555049

[13] National Marine Fisheries Service (NMFS). Fisheries of the United States, 2017 Report U.S. Department of Commerce, NOAA Current Fishery Statistics No. 2017 2018.

[14] Wells P. A quarantine surprise: Americans are cooking more seafood. The New York Times. 2020 May 5 [Cited 2020 July 22]. From: https://nyti.ms/3deyKHW

[15] Kuschner E. The future of fish is frozen: how the seafood industry is adapting to COVID-19. The Boston Globe. 2020 May 17 [Cited 2020 July 20]. From: https://www.boston.com/food/coronavirus/2020/05/17/seafood-industry-adapts-to-coronavirus

[16] Herz N. COVID-19 outbreak in Pacific Northwest seafood industry as season ramps up. National Public Radio. 2020 June 5 [Cited 2020 July 16]. Available from: https://www.npr.org/sections/coronavirus-live-updates/2020/06/05/870312092/pacific-northwest-seafood-industry-faces-covid-19-outbreak-as-season-ramps-up

[17] Smith Q. Pacific Seafood announces that 124 workers at its Newport plants have tested positive for COVID-19; closes all five facilities. Yachats Community News. 2020 June 7 [Cited 2020 July 20]. From: https://yachatsnews.com/pacific-seafood-announces-that-124-workers-at-its-newport-plants-have-tested-positive-for-covid-19-closes-all-five-facilities/

[18] Dandurant K. Fishermen selling from boat to table in response to COVID-19 challenges. Fosters.com. 2020 April 27. From: https://www.fosters.com/news/20200427/fishermen-selling-from-boat-to-table-in-response-to-covid-19-challenges

[19] Chesapeake Bay Magazine. Bay seafood sellers switch to direct sales amid pandemic. 2020 April 13. From: https://chesapeakebaymagazine.com/video-bay-seafood-sellers-shift-to-direct-sales-amid-pandemic/

[20] Drummond C. Weathering the storm: Rhode Island’s commercial fishery hit hard by COVID-19 pandemic. The Westerly Sun. 2020, April 6 [Cited 2020 July 16]. From: https://www.thewesterlysun.com/news/covid-19/weathering-the-storm-rhode-islands-commercial-fishery-hit-hardby-covid-19-pandemic/article_3305f058-783d-11ea-be3b-cf4ea09a73b5.html

[21] Stoll JS, Harrison HL, De Sousa E, Callaway D, Collier M, Harrell K, et al Alternative seafood networks during COVID-19: Implications for resilience and sustainability. EcoEvoRxiv Preprints. 2020, September 14. 10.32942/osf.io/kuzwq

[22] Belarmino EH, Bertmann F, Wentworth T, Biehl E, Neff R, Niles MT. The Impact of COVID-19 on the Local Food System: Early findings from Vermont. College of Agriculture and Life Sciences Faculty Publications. 23. 2020 Available from: https://scholarworks.uvm.edu/calsfac/23

[23] Coulthard S. Can we be both resilient and well, and what choices to people have? Incorporating agency into the resilience debate from a fisheries perspective. Ecology and Society. 2012; 17(1). 10.5751/ES-04483-170104

[24] Belhabib D, Dridi R, Padilla A, Ang M, Le Billon P. Impacts of anthropogenic and natural “extreme events” on global fisheries. Fish and Fisheries. 2018; 19(6), 1092–1109. 10.1111/faf.12314

[25] National Marine Fisheries Service. 2009 Fishing Communities of the United States, 2006. U.S. Dept. Commerce, NOAA Tech. Memo. NMFS-F/SPO-98, 84 p. Available at: http://www.st.nmfs.noaa.gov/st5/publication/index.html

[26] Colburn LL, Clay PM. The Role of Oral Histories in the Conduct of Fisheries Social Impact Assessments in Northeast US. Journal of Ecological Anthropology. 2012; 15(1): 74–80.

[27] Pollnac RB, Seara T, Colburn LL, Jepson M. Taxonomy of USA east coast fishing communities in terms of social vulnerability and resilience. Environmental Impact Assessment Review. 2015; 55: 136–143. 10.1016/j.eiar.2015.08.006

[28] Clay PM, Colburn LL, Seara T. Social bonds and recovery: An analysis of Hurricane Sandy in the first year after landfall. *Marine Policy*. 2016; 74: 334–340. 10.1016/j.marpol.2016.04.049

[29] Mills KE, Pershing AJ, Brown CJ, Chen Y, Chiang F-S, Holland, DS, et al Fisheries management in a changing climate: lessons from the 2012 heat wave in the Northwest Atlantic. Oceanography. 2013; 26(2): 191–195. https://www.jstor.org/stable/24862052

[30] Stoll JS, Crona BI, Fabinyi M, Farr ER. Seafood Trade Routes for Lobster Obscure Teleconnected Vulnerabilities. Front. Mar. Sci. 2018; 5:239 10.3389/fmars.2018.00239

[31] NOAA Fisheries. Fishery Disaster Determinations. N.d. Washington, DC [cited 2020 July 15]. Available from: https://www.fisheries.noaa.gov/national/funding-and-financial-services/fishery-disaster-determinations#numbers-72—54

[32] Colburn LL, Clay PM, Seara T, Weng C, Silva A. Social and Economic Impacts of Hurricane/Post Tropical Cyclone Sandy on the Commercial and Recreational Fishing Industries: New York and New Jersey One Year Later. NOAA Technical Memorandum NMFS-F/SPO-157. 2015: 68 p. Available online at: https://www.st.nmfs.noaa.gov/Assets/economics/documents/sandy/social-econ-hurricane-sandy.pdf

[33] Acheson J, Gardner R. Fishing failure and success in the Gulf of Maine: lobster and groundfish management. Maritime Studies. 2014; 13(8). http://www.maritimestudiesjournal.com/content/13/1/8

[34] Clay PM, Kitts A, Pinto da Silva P. Measuring the social and economic performance of catch share programs: Definition of metrics and application to the US Northeast Region groundfish fishery. Marine Policy. 2014; 44, 27–36. https://doi-org.proxy.libraries.rutgers.edu/10.1016/j.marpol.2013.08.009

[35] Clay PM, Olson J. Defining "Fishing Communities": Vulnerability and the Magnuson-Stevens Fishery Conservation and Management Act. Human Ecology Review. 2008; 15(2): 143–160.

[36] Berkes F. Understanding uncertainty and reducing vulnerability: Lessons from resilience thinking. Natural Hazards. 2007; 41(2): 283–295. 10.1007/s11069-006-9036-7

[37] Ferro-Azcona H, Espinoza-Tenorio A, Calderón-Contreras R, Ramenzoni VC, Gómez País MdlM, Mesa-Jurado MA. Adaptive capacity and social-ecological resilience of coastal areas: A systematic review. Ocean & Coastal Management. 2019; 173: 36–51. 10.1016/j.ocecoaman.2019.01.005

[38] Seara T, Pollnac R, Jakubowski K. Impacts of Natural Disasters on Subjective Vulnerability to Climate Change: A Study of Puerto Rican Fishers’ Perceptions after Hurricanes Irma & Maria. Coastal Management. 2020: 1–18. 10.1080/08920753.2020.1795969

[39] Schoon ML, Cox ME. Understanding Disturbances and Responses in Social-Ecological Systems. *Soc Nat Resour*. 2012; 25: 141–55. 10.1080/08941920.2010.549933

[40] Scyphers SB, Picou JS, Grabowski JH. Chronic social disruption following a systemic fishery failure. PNAS. 2019; 116(46): 22912–22914. www.pnas.org/cgi/doi/10.1073/pnas.1913914116 31659050 10.1073/pnas.1913914116 PMC6859345

[41] National Marine Fisheries Service. Fisheries Economics of the United States, 2016. U.S. Dept. of Commerce, NOAA Tech. Memo. NMFS-F/SPO-187. 2018; 243 p. Available from: https://www.fisheries.noaa.gov/resource/document/fisheries-economics-united-states-report-2016

[42] Henry A, Olson J. An overview of the survey on the socio-economic aspects of commercial fishing crew in the Northeast. NOAA Technical Memorandum NMFS-NE-230. 2014 9 From: https://repository.library.noaa.gov/view/noaa/4862

[43] R Core Team. R: A language and environment for statistical computing R Foundation for Statistical Computing, Vienna, Austria 2019 Available from: https://www.R-project.org/.

[44] Lüdecke D. _sjPlot: Data Visualization for Statistics in Social Science_. 2020 10.5281/zenodo.1308157 (URL: 10.5281/zenodo.1308157), R package version 2.8.3, <URL: https://CRAN.R-project.org/package=sjPlot>.

[45] Allaire JJ, Gandrud C, Russell K, Yetman CJ. networkD3: D3 JavaScript Network Graphs from R. R package version 0.4. 2017 From: https://CRAN.R-project.org/package=networkD3

[46] Fisheries NOAA. Greater Atlantic Regional Fisheries Office, Analysis and Program Support Division. Commercial landings 2015–2020, NMFS Dealer Weighout Database [dataset]. 23 July 2020 [Cited 28 July 2020].

[47] Johnson TR, Mazur M. A mixed method approach to understanding the graying of Maine’s lobster fleet. Bulletin of Marine Science. 2017; 94(3): 1185–1199. 10.5343/bms.2017.1108

[48] McClenachan L, Scyphers S, Grabowski JH. Views from the dock: warming waters, adaptation, and the future of Maine’s lobster fishery. Ambio. 2020; 49: 144–155. 10.1007/s13280-019-01156-3 30852777 PMC6889303

[49] U.S. Department of Agriculture (USDA). USDA announces additional food purchase plans. 2020 May 4 [Cited 2020 July 28]. From: https://www.ams.usda.gov/press-release/usda-announces-additional-food-purchase-plans

[50] Bittenbender S. SBA extends PPP loan eligibility to more fishing vessel owners. Seafood Source. 2020 June 26 [Cited 2020 July 29]. From: https://www.seafoodsource.com/news/business-finance/sba-expands-ppp-loan-eligibility-to-more-fishing-vessel-owners

[51] Stoll JS, Pinto da Silva P, Olson J, Benjamin S. Expanding the ‘geography’ of resilience in fisheries by bringing focus to seafood distribution systems. Ocean and Coastal Management. 2015; 116: 185–192. 10.1016/j.ocecoaman.2015.07.019

[52] Smith CL, Clay PM. Measuring subjective and objective well-being: analyses from five marine commercial fisheries. Human Organization. 2010; 69(2): 158–168.

[53] Smith TD. Scaling Fisheries: The science of measuring the effects of fishing, 1855–1955 Cambridge: Cambridge University Press 1994 10.1017/CBO9780511470868

[54] Seara T, Pollnac RB, Poggie JJ, Garcia-Quijano C, Monnereau I, Ruiz V. Fishing as therapy: Impacts on job satisfaction and implications for fishery management. Ocean and Coastal Management. 2017; 141, 1–9. 10.1016/j.ocecoaman.2017.02.016

[55] Thompson C, Johnson T, Hanes S. Vulnerability of fishing communities undergoing gentrification. Journal of Rural Studies. 2016; 45: 165–174. 10.1016/j.jrurstud.2016.03.008

[56] Crona BI, Basurto X, Squires D, Gelcich S, Daw TM, Khan A, et al Towards a typology of interactions between small-scale fisheries and global seafood trade. Marine Policy. 2016; 65: 1–10. 10.1016/j.marpol.2015.11.016

[57] Ramenzoni VC. Is Environmental Uncertainty Redefining Fishing Strategies? The Use of the Traditional Lunar Calendar to Allocate Fishing Effort in Ende, Eastern Indonesia In: Wood DC, editor. Climate Change, Culture, and Economics: Anthropological Investigations. Emerald Group Publishing Limited; 2015 Vol. 35, pp. 177–211. http://www.emeraldinsight.com/doi/abs/10.1108/S0190-128120150000035008

[58] van Oostenbrugge JAE, van Densen WLT, Machiels MAM. How the uncertain outcomes associated with aquatic and land resource use affect livelihood strategies in coastal communities in the central Moluccas, Indonesia. Agricultural Systems. 2004; 82(1): 57–91. 10.1016/j.agsy.2004.01.002

[59] van Densen WLT. On the perception of time trends in resource outcome: Its importance in fisheries co-management, agriculture and whaling Enschede: University of Twente; 2001. 299 p.

[60] van Oostenbrugge JAE. Uncertainty in daily catch rate in the light fisheries around Ambon and the Lease Islands: Characterisation, causes and consequences Doctoral Dissertation, Wageningen University. 2003 Available from: https://edepot.wur.nl/121496

[61] Wilson J. Learning, adaptation, and the complexity of human and natural interactions in the ocean. Ecology and Society. 2017; 22(2). https://doi.org/10/gbf8sq 29780429

[62] Perry RI, Ommer RE, Barange M, Jentoft S, Neis B, Sumaila UR. Marine social-ecological responses to environmental change and the impacts of globalization. Fish and Fisheries. 2011, 12(4): 427–450. 10.1111/j.1467-2979.2010.00402.x

[63] Witter A, Stoll JS. Participation and resistance: alternative seafood marketing in a neoliberal era. Marine Policy. 2017; 80: 130–140. https://doi-org.proxy.libraries.rutgers.edu/10.1016/j.marpol.2016.09.023

[64] Cumming G, Hunter-Thomson K, Young T. Local food 2.0: how do regional, intermediated, food value chains affect stakeholder learning? A case study of a community-supported fishery (CSF) program. Journal of Environmental Studies and Sciences. 2020; 10: 68–82. 10.1007/s13412-019-00577-6

[65] Aguilera SE, Cole J, Finkbeiner EM, Le Cornu E, Ban NC, Carr MH, et al Managing small-scale commercial fisheries for adaptive capacity: Insights from dynamic social-ecological drivers of change in monterey bay. PLoS ONE. 2015; 10(3). 10.1371/journal.pone.0118992 25790464 PMC4366077

[66] Cinner JE, Bodin Ö. Livelihood Diversification in Tropical Coastal Communities: A Network-Based Approach to Analyzing ‘Livelihood Landscapes’. PLoS ONE. 2010; 5(8): e11999 10.1371/journal.pone.0011999 20711442 PMC2920305

[67] Himes-Cornell A, Hoelting K. Resilience strategies in the face of short- and long-term change: Out-migration and fisheries regulation in Alaskan fishing communities. Ecology and Society. 2015; 20 10.5751/ES-07074-200209

[68] Daw TM, Cinner JE, McClanahan TR, Brown K, Stead SM, Graham NAJ, et al To Fish or not to Fish: Factors at multiple scales affecting artisanal fishers’ readiness to exit a declining fishery. PLoS ONE. 2012; 7(2). 10.1371/journal.pone.0031460 22348090 PMC3277441

[69] Johnson T, Henry AM, Thompson C. 2014. Qualitative indicators of social resilience in small-scale fishing communities: an emphasis on perceptions and practice. Human Ecology Review. 2017; 20(2): 97–115. Available from: 10.22459/HER.20.02.2014.05

[70] Pollnac RB, Poggie JJ. Happiness, well-being, and psychocultural adaptation to the stresses associated with marine fishing. Human Ecology Review. 2008; 15(2): 194–200.

[71] Myers HJ, Moore MJ. Reducing effort in the U.S. American lobster (Homarus americanus) fishery to prevent North Atlantic right whale (Eubalaena glacialis) entanglements may support higher profits and long-term sustainability. Marine Policy. 2020; 118 10.1016/j.marpol.2020.104017

[72] Wiedenmann J, Jensen OP. Uncertainty in stock assessment estimates for New England groundfish and its impact on achieving target harvest rates. Canadian Journal of Fisheries and Aquatic Sciences. 2018, 75(3): 342–256. 10.1139/cjfas-2016-0484

[73] Pinsky ML, Worm B, Fogarty MJ, Sarmiento JL, Levin SA. Marine taxa track local climate velocities. Science. 2013, 341: 1239–1242. 10.1126/science.1239352 24031017

[74] Bell RJ, Richardson DE, Hare JA, Lynch PD, Fratantoni PS. Disentangling the effects of climate, abundance, and size on the distribution of marine fish: an example based on four stocks from the Northeast US shelf. ICES J. Mar. Sci. 2015; 72 (5): 1311–1322. 10.1093/icesjms/fsu217

[75] Kleisner KM, Fogarty MJ, McGee S, Hare JA, Moret S, Perretti CT, et al Marine species distribution shifts on the U.S. Northeast Continental Shelf under continued ocean warming. Progress in Oceanography. 2017; 153: 24–36. 10.1016/j.pocean.2017.04.001

[76] Gephart JA, Deutsch L, Pace M, Troell M, Seekell D. Shocks to fish production: identification, trends, and consequences. Global Environmental Change. 2017; 42: 24–32. 10.1016/j.gloenvcha.2016.11.003

[77] National Oceanic and Atmospheric Administration (NOAA), National Marine Fisheries Service. Magnuson-Stevens Fishery Conservation and Management Act. U.S. Public Law No. 109–479. (2007). https://www.fisheries.noaa.gov/resource/document/magnuson-stevens-fishery-conservation-and-management-act

