# Supplementary material for: Adaptation and resilience of commercial fishers in the Northeast United States during the early stages of the COVID-19 pandemic
Source: PLoS One. 2020 Dec 17;15(12):e0243886. doi: 10.1371/journal.pone.0243886 (PMC7746300; doi:10.1371/journal.pone.0243886)
Supplement: S1 File — (PDF) [file pone.0243886.s001.pdf]

Default Question Block

Welcome to the COVID-19 Fisheries Livelihoods Survey

CONSENT TO TAKE PART IN ANONYMOUS RESEARCH

TITLE OF STUDY: Adapting to changes in "fishing opportunity portfolios": abundance, availability, and access. Extension: Impacts of COVID-19 on local fisheries.

Principal Investigator: Victoria C. Ramenzoni, Ph.D.

This consent form is part of an informed consent process for a research study and it will provide information that will help you decide whether you want to take part in this study. It is your choice to take part or not. Your alternative to taking part in the research is not to take part in it.

Who is conducting the study and what is it about?
You are invited to take part in a research study that is being conducted by Dr. Victoria Ramenzoni, who is a professor in the Department of Human Ecology at Rutgers University. The purpose of the research is to improve our understanding of socioeconomic and climate change pressures affecting Northeast US fishing communities, including the COVID-19 pandemic, in order to aid fisheries managers, planners, and decision makers develop better tools to address future threats associated with socioeconomic and environmental change. We are conducting surveys with fishermen throughout the Northeast U.S. and would like to know more about your experience, the effects of the current pandemic crisis on fishing, and impacts you may have suffered from social distancing measures, and the ways in which you may be adapting to this crisis.

Dr. Ramenzoni may be reached at:
Email: victoria.ramenzoni@rutgers.edu

Phone: (848) 932-9153

Department of Human Ecology, Rutgers University

55 Dudley Road

New Brunswick, New Jersey 08901

What will I be asked to do if I take part in the study?

You will be asked to answer several questions about your recent experiences with fisheries and about changes to your fishing practices since the start of the COVID-19 pandemic. The information will be anonymously collected. No one will know which responses are yours. Your participation in the study will be about 15-20 minutes. We anticipate 250 subjects will take part in the study.

What are the risks of harm or discomforts I might experience if I take part in the study?

There are minimum risks with participating in this study. Risks may be related to experiencing some level of discomfort while answering survey questions. You can decline to answer any questions that you may find not suitable without any repercussions.

Are there any benefits to me if I choose to take part in this study?

There are no immediate benefits that you will obtain from taking part of this study. The benefits of taking part in this study may be related to increasing our knowledge of the effects of socioeconomic, environmental, and public health-related pressures on fishing activities in the region. This will help local decision-makers and managers better address these impacts and develop mitigative solutions.

Will I be paid to take part in this study?

You will not be paid to take part in this study.

How will information about me be kept private or confidential?

The research is anonymous. No information will be collected that can identify who you are. Additionally, to keep the data safe, data will be encrypted and/or password protected. Numerical identifiers and data keys will be deleted after data analysis and manuscript development is complete. Only the main investigators and associated researchers will have access to the raw data files and encryption keys. All data will be stored safely in the main researcher's office or in encrypted and password protected websites. Study data will be kept for 2 years after the completion of the study.

What will happen to information I provide in the research after the study is over?
After the study is over the information may be used by or distributed to investigators for other research without obtaining additional permission from you.

The research team and the Institutional Review Board at Rutgers University are the only parties that may see the data, except as may be required by law. If the findings of this research are professionally presented or published, only group results will be stated.

What will happen if I do not wish to take part in the study or I later decide not to stay in the study?
It is your choice whether you take part in the research. You may choose to take part, not to take part or you may change your mind and withdraw from the study at any time. In addition, you can choose to skip questions that you are not comfortable answering. If you do not want to enter the study or decide to stop taking part, your relationship with the study staff will not change, and you may do so without penalty and without loss of benefits to which you are otherwise entitled. Please note, however, that once you have submitted your responses, you may no longer withdraw them as we will not know which ones yours are.

If you have questions about taking part in this study, you can contact the Principal Investigator:
Dr. Ramenzoni, Department of Human Ecology, Rutgers University
Email: victoria.ramenzoni@rutgers.edu
Phone: (848) 932-9153

If you have questions about your rights as a research subject, you can contact the IRB Director at:
New Brunswick/Piscataway Arts and Sciences IRB (732) 235-2866 or the Rutgers Human Subjects Protection Program at (973) 972-1149 or email us at humansubjects@ored.rutgers.edu.

By beginning this research, I acknowledge that I am 18 years of age or older and have read and understand the information. I agree to take part in the research, with the knowledge that I am free to withdraw my participation in the research without penalty.

Please print a copy of this consent form for your records.

If you are 18 years of age or older, understand the statements above, and consent to take part in the study, select "I Agree" to begin the survey. If not, please select “I Do

Not Agree".

I Agree ◯ | I Do Not Agree ◯

What is your age?

What is your gender?

What is your Zip Code?

What ethnicity do you most identify with?

- ◯ Hispanic/ Latino
- ◯ Black/ African American
- ◯ Asian
- ◯ Native Hawaiian/ Pacific Islander
- ◯ Native American
- ◯ Caucasian/ White
- ◯ Middle Eastern
- ◯ Multi-Racial
- ◯ Other

Highest level of education achieved:

- ◯ Did not graduate high school
- ◯ Graduated high school or earned a GED

- Attended some college but did not graduate
- Graduated college with an Associate's degree
- Graduated college with a Bachelor's degree
- Attended some graduate school but did not graduate with an advanced degree (Master's, PhD, MD, etc.)
- Graduated with an advanced degree (Master's, PhD, MD, etc.)

Marriage status:

- Single
- Married
- Living with a partner
- Divorced/ separated
- Widowed

Which category best describes your residence/ living arrangement?

- Own home
- Rent home
- Living in housing provided by employer
- Live in a house but do not pay rent
- Other:

How many people are in your household?

How many years have you been involved in commercial fisheries?

How would you describe your main role currently?

- Owner
- Captain
- Crew

How many years have you been in this role?

Home port:

Landing port(s):

Port 1

Port 2

Port 3

Port 4

Port 5

Number of vessels you own or work on:

Size of vessel(s) (check all that apply):

- Less than 30 feet
- 30-49 feet
- 50-74 feet
- 75 feet or greater

Type(s) of gear (check all that apply):

- ☐ Otter trawl
- ☐ Gillnet
- ☐ Scallop dredge
- ☐ Lobster/ crab pots
- ☐ Fish pots
- ☐ Pelagic longline
- ☐ Handline
- ☐ Hook and line/tub trawl
- ☐ Mid-water trawl
- ☐ Clam dredge
- ☐ Bull rake
- ☐ Conch/whelk pots
- ☐ Other: ____________

What fisheries do you typically participate in? Please list, separating your answer(s) with a comma:

What species do you typically fish for? Please list, separating your answer(s) with a comma:

What would you normally be fishing for at this time of year? Please list, separating your answer(s) with a comma:

What permits do you currently hold? Please list, separating your answer(s) with a comma:

For Captains/Owners - How would you describe the state of your fishing income over the last five years, **prior to the outbreak of the coronavirus:**

- My income from fishing had been increasing significantly (an increase of more than 20%)
- My income from fishing had been increasing slightly (an increase of 5-20%)
- My income from fishing had remained steady (increase or decrease of no more than 5%)
- My income from fishing had been decreasing slightly (a decrease of 5-20%)
- My income from fishing had been decreasing significantly (a decrease of more than 20%)

Have you continued fishing since social distancing restrictions went into place?

- Yes
- No

If yes, what species are you currently targeting? Please list, separating your answer(s) with a comma:

Since social distancing measures went into effect in your state, has your income from fisheries:

- Been much higher (greater than 20%) than the past 3 years
- Been somewhat higher (5-20%) than the past 3 years
- Been about the same (within 5%)
- Been somewhat lower (5-20%) than the past 3 years
- Been much lower (greater than 20%) than the past 3 years
- I'm not earning any income from fishing right now
- Not sure

Since social distancing measures went into effect in your state, have your landings:

- Been much higher (greater than 20%) than the past 3 years
- Been somewhat higher (5-20%) than the past 3 years
- Been about the same (within 5%)
- Been somewhat lower (5-20%) than the past 3 years
- Been much lower (greater than 20%) than the past 3 years
- I'm not earning any income from fishing right now
- Not sure

Since social distancing measures went into effect in your state, has the number of fishing trips you've taken:

- Been much higher (greater than 20%) than the past 3 years
- Been somewhat higher (5-20%) than the past 3 years
- Been about the same (within 5%)

- Been somewhat lower (5-20%) than the past 3 years
- Been much lower (greater than 20%) than the past 3 years
- I'm not earning any income from fishing right now
- Not sure

Since social distancing measures went into effect in your state, has the amount of time you've spent fishing:

- Been much higher (greater than 20%) than the past 3 years
- Been somewhat higher (5-20%) than the past 3 years
- Been about the same (within 5%)
- Been somewhat lower (5-20%) than the past 3 years
- Been much lower (greater than 20%) than the past 3 years
- I'm not earning any income from fishing right now
- Not sure

Since social distancing measures went into effect in your state, have the overall costs of fishing (including fuel, ice, supplies, etc.):

- Been much higher (greater than 20%) than the past 3 years
- Been somewhat higher (5-20%) than the past 3 years
- Been about the same (within 5%)
- Been somewhat lower (5-20%) than the past 3 years
- Been much lower (greater than 20%) than the past 3 years
- I'm not earning any income from fishing right now
- Not sure

For each of your typical fishing costs, please indicate whether they have increased, decreased, or stayed the same:

	Increased	Decreased	Stayed about the same	N/A
Fuel	○	○	○	○
Crew Wages/Share	○	○	○	○
Ice	○	○	○	○
Bait	○	○	○	○
Repairs	○	○	○	○
Trip supplies (food, water, etc.)	○	○	○	○

How many crew do you usually carry on board at this time of year?

How many crew are you carrying right now on a typical trip?

Are your crew members:

- ○ Employees
- ○ Independent contractors (self-employed)
- ○ Family members
- ○ A mix
- ○ Others

If you've had to let go of any crew members because of the pandemic, have they been able to take advantage of state or federal relief programs (such as unemployment assistance or PPP loans):

- ○ Yes
- ○ No
- ○ Don't know

○ N/A

How have your fishing income and activity been affected by the COVID-19 pandemic and its impacts? Please indicate how significant the impact of the following effects has been to your normal fishing activities:

	No impact 0	Very little impact 1	Little impact 2	Some impact 3	Significant impact 4	Very significant impact 5
Loss of export markets	○	○	○	○	○	○
Loss of domestic markets	○	○	○	○	○	○
Loss of restaurant sales	○	○	○	○	○	○
Decreasing price for seafood products	○	○	○	○	○	○
Challenges trying to obey social distance guidelines (staying six feet apart, wearing masks) while on a boat	○	○	○	○	○	○
Unable to take enough crew fishing because of social distancing guidelines	○	○	○	○	○	○
Illness or concerns about illness (of you or crew members) due to COVID-19	○	○	○	○	○	○
Can't hire enough crew because of labor shortages	○	○	○	○	○	○
Crew members are leaving because of the availability of unemployment benefits	○	○	○	○	○	○
Loss of processing capacity (processors closing, or having insufficient numbers of workers)	○	○	○	○	○	○
Lack of ice	○	○	○	○	○	○
Lack of bait	○	○	○	○	○	○
Lack of fuel	○	○	○	○	○	○

	No impact 0	Very little impact 1	Little impact 2	Some impact 3	Significant impact 4	Very significant impact 5
Can't get vessel repairs or gear repairs	○	○	○	○	○	○
Need to change dealers or shift the location or timing of landings because of dealer requirements	○	○	○	○	○	○
Other: ____	○	○	○	○	○	○

Please describe other ways in which your business or livelihood has been affected by the coronavirus pandemic and the impacts of social distancing:

In what ways have you adapted to the new circumstances of the coronavirus pandemic? Check all that apply:

- ☐ Participating in direct sales of seafood to customers
- ☐ Participating in seafood delivery to customers
- ☐ Participating in a CSF or other marketing program (please describe): ____
- ☐ Fishing for different species (please describe): ____
- ☐ Fishing in a different location or changing trip lengths (please describe): ____

☐ Switching to or supplementing with a non-fishing source of income (please describe): ________

☐ I am no longer fishing

☐ I have not changed my business to adapt to the coronavirus pandemic

Have you received or do you expect to receive any kind of support from the government since the start of the pandemic? If so, check all that apply:

☐ Receiving fishery disaster assistance from NOAA through the CARES Act (Section 12005 funds)

☐ Receiving Pandemic Unemployment Assistance

☐ Economic Impact Payment (Stimulus Check)

☐ Small Business Association (SBA) Economic Injury Disaster Loan

☐ Paycheck Protection Program (PPP) Loan

☐ IRS Special Exemption

☐ Other: ________

If you have received funds, please indicate your degree of satisfaction with the amount of the payment(s):

○ Extremely satisfied

○ Moderately satisfied

○ Slightly satisfied

○ Neither satisfied nor dissatisfied

○ Slightly dissatisfied

○ Moderately dissatisfied

○ Extremely dissatisfied

○ I have not received funds

If you have received funds, please indicate your degree of satisfaction with the amount of time it took to receive payment(s).

- Extremely satisfied
- Somewhat satisfied
- Neither satisfied nor dissatisfied
- Somewhat dissatisfied
- Extremely dissatisfied
- I have not received funds

Since the coronavirus pandemic began, has working as a fisherman become:

- Much more stressful than normal
- Somewhat more stressful than normal
- About as stressful as normal
- Somewhat less stressful than normal
- Much less stressful than normal
- I am not fishing

Please indicate the extent to which you or any immediate family member(s) have experienced the following problems directly related to the COVID-19 crisis:

	Not at all			Somewhat			Very much
	1	2	3	4	5	6	7
Immediate health problems directly related to the crisis.	○	○	○	○	○	○	○
Immediate mental health issues such as anxiety or depression directly related to the crisis.	○	○	○	○	○	○	○

	Not at all 1	2	3	Somewhat 4	5	6	Very much 7
Ongoing health problems such as anxiety or depression.	○	○	○	○	○	○	○
Immediate financial problems from loss of work directly related to the crisis.	○	○	○	○	○	○	○
Immediate financial problems directly related to the prevention of COVID-19 to maintain social distancing (increasing expenditures in groceries, cleaning products, etc.).	○	○	○	○	○	○	○
Immediate financial problems due to medical bills for treatment of COVID-19.	○	○	○	○	○	○	○

Please indicate to what extent:

	Changed for the worse 1	2	3	No Change 4	5	6	Changed for the better 7
Your health promoting behaviors (for example exercise, diet, etc.) have changed due to COVID-19.	○	○	○	○	○	○	○
Your health impairing behaviors (for example smoking, alcohol use) have changed due to COVID-19.	○	○	○	○	○	○	○
Your stress management behaviors (for example seeking support) have changed due to COVID-19.	○	○	○	○	○	○	○

	Changed for the worse 1	2	3	No Change 4	5	6	Changed for the better 7
Your access to health care (for example seeing a health care provider, obtaining medications) is affected by the crisis.	○	○	○	○	○	○	○
Your ability to access mental health care has changed due to the crisis.	○	○	○	○	○	○	○
The availability of other people in your community who could help you.	○	○	○	○	○	○	○

How confident do you feel that you will still be fishing in 3 years from now?

- ○ I am very confident I will still be fishing
- ○ I am somewhat confident I will still be fishing
- ○ I am not sure if I will still be fishing
- ○ I am somewhat confident I will no longer be fishing
- ○ I am very confident I will no longer be fishing
- ○ I have plans to retire from fishing before that time

If your fishing has been affected by the pandemic, when do you expect your normal fishing activity will resume?

- ○ Within the next 3 months
- ○ Within the next 6 months
- ○ Within the next 12 months
- ○ Within the next 2 years
- ○ Longer than 2 years
- ○ Fishing has already resumed normally or has not changed
- ○ Never

- Don't know

How much of your income do you normally derive from fishing?

- Less than 25%
- 25-49%
- 50-74%
- 75-99%
- I derive 100% of my income from fishing

Do you have alternative sources of income other than fishing?

- No
- Yes, please describe: ____________

What is your household's approximate annual income?

- Less than $20,000
- $20,000 to $40,000
- $41,000 to $60,000
- $61,000 to $80,000
- $81,000 to $100,000
- $101,000 to $120,000
- Over $120,000

Would you be willing to participate in a follow-up interview about the impacts of the coronavirus pandemic on your livelihood? If so, please provide your contact information and we may follow up with you:

Name:

Phone number:

Email address:

Thank you for your participation in the survey.

Please click the next arrow to complete your submission.

Powered by Qualtrics
