# Supplementary material for: Adaptation and resilience of commercial fishers in the Northeast United States during the early stages of the COVID-19 pandemic
Source: PLoS One. 2020 Dec 17;15(12):e0243886. doi: 10.1371/journal.pone.0243886 (PMC7746300; doi:10.1371/journal.pone.0243886)
Supplement: S1 Fig — The left-hand nodes indicate the species or groups of species that respondents reported normally targeting at the time of year at which the survey was administered (May-June). The right-hand nodes indicate the species or species groups they reported currently targeting at the time of the survey. Vertical height of each node denotes the proportion of all respondents who answered species-specific questions selecting that species or species groups. Horizontal links between the same species indicate the proportion of fishers who habitually target that species and continued fishing for it during the pandemic. Horizontal links between different species indicate the proportion of fishers who switched from the species on the left to the species on the right. The “no longer fishing” node indicates the proportion of fishers who stopped fishing entirely this spring. NE = New England; MA = Mid-Atlantic; HMS = Highly Migratory Species. See Table 1 for fisheries group definitions. Note that proportions do not equal 1, as many fishers targeted multiple species. (HTML) [file pone.0243886.s002.html]

sankeyNetwork
