# Supplementary material for: Adaptation and resilience of commercial fishers in the Northeast United States during the early stages of the COVID-19 pandemic
Source: PLoS One. 2020 Dec 17;15(12):e0243886. doi: 10.1371/journal.pone.0243886 (PMC7746300; doi:10.1371/journal.pone.0243886)
Supplement: S1 Table — (PDF) [file pone.0243886.s004.pdf]

State	Date Stay-at-Home orders began	Date Stay-at-Home orders lifted
Connecticut	March 23	May 20
Delaware	March 24	May 31
Maine	April 2	June 1 (*stores and restaurants were allowed to open sooner in counties without community spread)
Maryland	March 30	May 15
Massachusetts (Stay-at-home advisory only)	March 23	May 18
New Hampshire	March 27	June 15 (restaurants opened sooner)
New Jersey	March 21	June 9
New York	March 22	May 28
North Carolina	March 30	May 22
Rhode Island	March 28	May 8
Virginia	March 30	June 10
