# Supplementary material for: Adaptation and resilience of commercial fishers in the Northeast United States during the early stages of the COVID-19 pandemic
Source: PLoS One. 2020 Dec 17;15(12):e0243886. doi: 10.1371/journal.pone.0243886 (PMC7746300; doi:10.1371/journal.pone.0243886)
Supplement: S2 Table — (DOCX) [file pone.0243886.s005.docx]

| **Gender** | **Number of Responses** |
| --- | --- |
| Male | 228 |
| Female | 10 |
| NA | 21 |
| Other/ Prefer not to say | 1 |
