# Supplementary material for: Adaptation and resilience of commercial fishers in the Northeast United States during the early stages of the COVID-19 pandemic
Source: PLoS One. 2020 Dec 17;15(12):e0243886. doi: 10.1371/journal.pone.0243886 (PMC7746300; doi:10.1371/journal.pone.0243886)
Supplement: S3 Table — (DOCX) [file pone.0243886.s006.docx]

| **What ethnicity do you most identify with?** | **Number of responses** |
| --- | --- |
| Caucasian/ White | 233 |
| Hispanic / Latino | 0 |
| Black/ African American | 0 |
| Asian | 2 |
| Native Hawaiian/ Pacific Islander | 0 |
| Native American | 0 |
| Middle Eastern | 0 |
| Multi-Racial | 1 |
| Other | 3 |
| NA | 21 |
