# Supplementary material for: Adaptation and resilience of commercial fishers in the Northeast United States during the early stages of the COVID-19 pandemic
Source: PLoS One. 2020 Dec 17;15(12):e0243886. doi: 10.1371/journal.pone.0243886 (PMC7746300; doi:10.1371/journal.pone.0243886)
Supplement: S4 Table — (DOCX) [file pone.0243886.s007.docx]

| **Fishery** | **Species included** | **Gear types included** | **Modal vessel size** | **Modal annual household income** |
| --- | --- | --- | --- | --- |
| *Marine benthic fish* | Northeast groundfish species (cod, haddock, flounders, pollock, redfish, halibut), summer flounder, black sea bass, tautog, hake, whiting, skates, monkfish, spiny dogfish, smooth dogfish, golden tilefish, blueline tilefish | Otter trawl, Gillnet, Handline, Hook and line/tub trawl, fish pots, fish weirs | 30-49 feet (n=66) | Bimodal: $81,000 to $100,000 (n=22) and More than $120,000 (n=22) |
| *Marine small pelagics* | Longfin squid, shortfin (illex) squid, butterfish, mackerel, Atlantic herring, shrimp | Otter trawl, mid-water trawl, purse seine | 75 feet or greater (n=15) | Bimodal: $100,000 - $120,000 (n=6) and Over $120,000 (n=6) |
| *Marine large pelagics/ highly migratory species* | Spanish mackerel, King mackerel, dolphin/mahi, wahoo, sharks, little tunny, bonito, albacore tuna, yellowfin tuna, bluefin tuna, bigeye tuna, swordfish | Rod and reel, pelagic longline, handline, hook and line/tub trawl, harpoon | 30-49 feet (n=22) | $81,000 - $100,000 (n=9) |
| *Marine bivalves* | Sea scallops, ocean quahogs, surf clams | Scallop dredge, clam dredge | 30-49 feet (n=22) | $41,000 - $60,000 (n=7) |
| *Marine crustaceans* | Lobster, Jonah crab, rock crab, red crab | Lobster / crab pots | 30-49 feet (n=116) | Over $120,000 (n=22) |
| *Coastal finfish* | Bluefish, striped bass, menhaden, sea trout/speckled trout, spot, croaker, drum, weakfish, toadfish, sea mullet, blowfish, American shad, baitfish, silversides, gudgeon, minnows | Gillnet, fish pots, handline, cast net, seine net, pound net, drop net, fyke net | Less than 30 feet (n=40) | $61,000 - $80,000 (n=13) |
| *Coastal invertebrates* | Shellfish (oysters, steamers/soft clams, sea urchins, quahogs/hard clams, razor clams, bay scallops, periwinkles), blue crabs, green crabs, conch, whelk, horseshoe crabs, starfish | Crab pots, dredge, bull rake, oyster dredge, crab dredge, conch/whelk pots, oyster tongs | Less than 30 feet (n=45) | $81,000 - $100,000 (n=12) |
| *All fisheries* |  |  | 30-49 feet (n=156) | $81,000 - $100,000 (n=40 / 19.4%) |
